# Dynamics of a goshawk population across half a century is driven by the variation of first‐year survival

**DOI:** 10.1002/ece3.70058

**Published:** 2024-08-01

**Authors:** Michael Schaub, Volkher Looft, Floriane Plard, Jan A. C. von Rönn

**Affiliations:** ^1^ Swiss Ornithological Institute Sempach Switzerland; ^2^ Honigkamp 20 Postfeld Germany; ^3^ Barraque de la Pinatelle, Tremoulet Molompize France

**Keywords:** *Accipiter gentilis*, density dependence, elasticity, females, integrated population model, males, retrospective population analysis, two sexes

## Abstract

Population dynamics are driven by stochastic and density‐dependent processes acting on demographic rates. Individuals differ demographically, and to capture these differences, models of population dynamics are usually structured by age and stage, rarely by sex. An effect of sex on population dynamics is expected if the dynamics of males and females differ, requiring an unequal sex ratio at birth and/or sex‐specific survival probabilities. Goshawks (*Accipiter gentilis*) show large sexual size dimorphism and differential survival, but it is unknown whether males and females contribute differently to population dynamics. We studied a goshawk population in northern Germany over 47 years using brood monitoring data, collected feathers and nestling ringing data. We jointly analyzed the data using a two‐sex integrated population model and performed retrospective and prospective population analyses to understand whether the demographic drivers of population change differ between the sexes. The population showed large fluctuations, during which the number of breeding pairs doubled, but the long‐term trend of the population was slightly negative. Female survival exceeded male survival during the first year of life. Females started to reproduce at a younger age than males, productivity increased with female age, the sex ratio of nestlings was male biased and there was moderate male immigration. Despite these differences, temporal variation in sex ratio did not contribute to population dynamics and the contribution of temporal variation in survival was similar for both sexes. Variation in first‐year survival was the strongest driver in this population, regulated by a weak density‐dependent feedback acting through female first‐year survival. Overall, the contributions of the two sexes to population dynamics were similar in this monogamous species with strong sexual size dimorphism.

## Graphical abstract

Temporal variation in first‐year survival was the strongest driver in the goshawk population studied, regulated by a weak density‐dependent feedback acting through female first‐year survival. Although the two sexes differed demographically, their contributions to population dynamics were similar in this monogamous species with strong sexual size dimorphism.

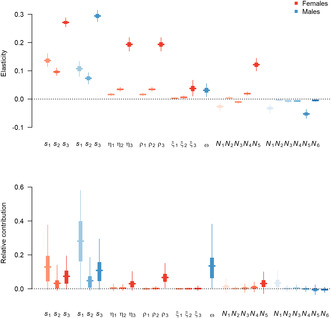

## 1 INTRODUCTION

The temporal change in the number of individuals in a population is the result of the individual demographic processes of survival, reproduction and dispersal. Individual processes are usually averaged at the population level into demographic rates. An understanding of population dynamics then focuses on how much potential future changes in demographic rates affect population dynamics, and how much past temporal variation in demographic rates has contributed to the observed dynamics of a population (Caswell, 2000). Since demographic rates play a key role in population dynamics, the way they are generated from individual demographic processes is important for inference. Individuals differ and their performance change with age or stage (Benton et al., 2006). Therefore, age or stage structure is usually included in demographic rates and population models.

Another way of classifying individuals is by sex. Although offsprings are produced by females, offspring production is dependent on males to varying degrees, and survival and dispersal may differ between the sexes. As a result, the dynamics of males and females may differ resulting in biased adult sex ratios with consequences on population growth (Rankin & Kokko, 2007) or the mating system (Heinsohn et al., 2019). To understand such populations, both sexes must be included and explicitly modeled in population models (Gerber & White, 2014; Lindström & Kokko, 1998). Although sex differences in survival have been found in all classes of vertebrates (Donald, 2007; Honeycutt et al., 2019; Lemaître et al., 2020; Sultanova et al., 2023; Székely et al., 2014) and non‐monogamous mating systems are widespread (Clutton‐Brock, 1989; Emlen & Oring, 1977; Møller, 1986), the application of single‐sex population models, which assume no sex differences in survival and monogamous mating systems, still dominates empirical population studies. Insights based on single‐sex population models can lead to misleading inferences, and in general there is little empirical knowledge of how strongly population dynamics are driven by the demography of each sex (but see e.g., Cleasby et al., 2017; Eberhart‐Phillips et al., 2017; Sawada et al., 2021). A possible reason for this gap is the increased data requirements, as demographic rates have to be estimated for both sexes. Sex differentiation is impossible in many species without genetic or other invasive methods, which is an additional major barrier to sex‐specific modeling.

The goshawk (*Accipiter gentilis*) is a medium‐sized woodland raptor with large sexual size dimorphism, with females being about 30% larger than males. Although it is monogamous, the two sexes have different roles in brood rearing: females incubate the eggs and brood the young while males hunt and provide food for the female and her offspring. The nestling sex ratio is usually male‐biased (Rutz, 2012; Ryttman, 2001) and survival often differs between the sexes, with male survival being lower than female survival (Kenward et al., 1999; Tornberg et al., 2006). Male and female dynamics would therefore be expected to differ, but to date the population dynamics of goshawks have mainly been studied using single sex population models (Krüger, 2007; Rutz, Bijlsma, et al., 2006). The population size of goshawks tends to fluctuate little (Rutz & Bijlsma, 2006), indicating strong density dependence (Krüger & Lindström, 2001; Rutz, Bijlsma, et al., 2006).

Here we study the dynamics of a goshawk population from Schleswig‐Holstein, Germany, over almost half a century. Using an integrated population model (Schaub & Abadi, 2011; Schaub & Kéry, 2022), we estimated sex‐ and age‐specific demographic rates and stage‐specific population sizes. We estimated the sex ratio of nestlings and included this parameter into our two‐sexes population model. We then assessed the sensitivity of the population growth rate to changes in the underlying demographic rates and performed a retrospective analysis to determine how much the demographic rates of each sex have contributed to the dynamics of this goshawk population. Since most demographic population studies on goshawk considered females only (Rutz, Bijlsma, et al., 2006), it is unclear whether the two sexes impact population dynamics differentially, as can be expected if the nestling sex ratio is uneven and survival sex‐specific. Finally, we assessed whether the goshawk population was limited by density dependent processes, and if so, through which demographic pathway they operated and whether sexes differed. Thereby, our study adds understanding in the role of the sexes on the dynamics of populations.

## 2 MATERIALS AND METHODS

### 2.1 Study species

The goshawk is widespread in Eurasia and North America preying mainly on birds (pigeons, grouse) and mammals (squirrels, hares; Glutz von Blotzheim et al., 1989). The large sexual size dimorphism has consequences for prey selection, females take larger prey than males (Glutz von Blotzheim et al., 1989), and females can build up more body reserves, making them less sensitive to starvation than males (Kenward et al., 1999; Sunde, 2002). The population dynamics of goshawks is characterized by high stability (Rutz & Bijlsma, 2006) and strong density dependence (Krüger & Lindström, 2001; Rutz, Bijlsma, et al., 2006). Productivity is spatially and temporally highly variable (Reynolds et al., 2017), and it increases with parental age (Nielsen & Drachmann, 2003; Risch et al., 2004) and food availability (Byholm, Ranta, et al., 2002; Ranta et al., 2003; Tornberg et al., 2006). The proportion of male nestlings increases with increasing food availability and brood size, and decreases seasonally (Byholm, Brommer, & Saurola, 2002; Byholm, Ranta, et al., 2002). Survival increases with age (Kenward et al., 1999; Krüger, 2005; Ryttman, 1993), and some studies found lower first‐year survival of males compared to females (Kenward et al., 1999), while others found no difference between sexes (Reynolds et al., 2017; Tolvanen et al., 2017). Reproducing goshawks are very philopatric to their breeding site (Byholm et al., 2003; Tolvanen et al., 2017; Tornberg et al., 2006) and natal dispersal is weak (Reynolds et al., 2017) with males dispersing further than females (Byholm et al., 2003).

### 2.2 Study area

The study was carried out in northern Germany (Appendix 1, Figure A‐1 in Supporting Information; Schleswig‐Holstein, 54.21°–54.73° N, 8.98°–9.65° E) in an area of 2000 km^2^ (Looft, 2017). The flat landscape is dominated by agricultural land (75% of the surface), urban areas (12%) and interspersed, mostly small forests (10%). Although the forests have been logged, their distribution and size remained constant over the study period. However, there have been major changes in the agricultural land, which used to be dominated by grassland and is now dominated by arable land (Looft, 2017).

### 2.3 Data collection

Fieldwork was carried out mainly from March to June each year from 1968 to 2014. Goshawk territories were visited at the end of March, before the broadleaved trees budded, and nest occupation was assessed by observing displaying adults, warning calls, droppings, molted feathers and prey remains. Territories occupied in previous years were visited first, and if there was no sign of goshawk presence, surrounding woodlands larger than 2 ha were searched for occupied nests. All consecutive visits to occupied nest sites were used to assess breeding success. In June, all occupied nests were climbed to mark nestlings with individually numbered rings from the Helgoland ringing scheme and to determine their sex (based on size, Bijlsma, 1994) and age (in days, based on juvenile plumage development, Bijlsma, 1994).

The mostly complete post‐juvenile (second calendar year) and post‐breeding (≥third calendar year) molt begins in April or May (Baker, 2016). During this period, females remain on their nests to incubate the eggs or in close proximity to protect the brood from predators, while males are more mobile while hunting to provide food for the female and the young in the early nestling stages. During nest visits, we searched intensively for and collected goshawk feathers in the nest and in a 100 m radius around the nest. The collected primary feathers were used to determine the age (3 levels: 1 year old (second calendar year), 2 years old (third calendar year) and at least 3 years old (fourth calendar year or older)) and the sex (feather size) of breeding birds, and to identify individuals. The identification of individual goshawks from multiple patterns on shed primary feathers is a reliable method for tracking individuals over their lifetime (Bezzel et al., 1997; Krüger, 2005, 2007; Kühnapfel & Brune, 1995; Nielsen & Drachmann, 2003; Opdam & Müskens, 1976). The high accuracy of the method to identify individuals over multiple years has been confirmed using a capture‐recapture approach (Ziesemer, 1983) and molecular genetic markers (Hoy et al., 2016; Selås et al., 2017).

From the field visits we compiled the following seven data sets: (1) number of occupied territories (*n* = 47 years), (2) sex‐ and age‐specific (3 age classes) annual number of breeders (based on the collected feathers), (3) success (successful vs. failed) of 1851 broods, (4) number of fledglings of successful broods (*n* = 1334 broods), (5) fledgling sex ratio (*n* = 1281 broods), (6) capture‐recapture data based on the collected feathers from 462 adult individuals (341 females, 121 males), and (7) 319 (137 females, 182 males) dead‐recoveries from a total of 3509 (1595 females, 1911 males) ringed nestlings. Most of the dead‐recoveries were reported by members of the public.

### 2.4 Data analysis

We jointly analyzed the seven data sets with an integrated population model (IPM; Schaub & Kéry, 2022) to estimate demographic rates and population sizes and to perform prospective and retrospective population analyses. The core of an IPM is a stage‐structured population model (Caswell, 2001) which links demographic rates and population size and which we describe in the following. All complete description of the IPM including the likelihoods of the seven data sets and of the joint likelihood which composes the IPM is given in Appendix 2 in Supporting Information.

We defined a pre‐breeding survey, two‐sexes stochastic model with multiple stages for each sex. Stage classes 1 and 2 refer to individuals that are 1‐year old and either breeding or non‐breeding, respectively, stage classes 3 to 4 to 2‐year‐old individuals that are also either breeding or non‐breeding, respectively, and stage class 5 refers to breeding individuals that are 3‐year‐old or older. Stage class 6 only occurs in males and includes immigrants (but see a model with additional female immigration in Appendix 3 in Supporting Information). We assume that all individuals 3‐years old or older are breeding. The stage‐specific numbers of individuals in year *t* + 1 are functions of the numbers in year *t* and the demographic rates. To account for demographic stochasticity, we use Poisson and binomial distributions.

The stage‐specific numbers of females in year *t* + 1 is modeled as,

$$\begin{array}{c} N_{1,f,t+1}∼\text{Poisson}\left(\left(N_{2,f,t}\eta_{1,t}\rho_{1,t}\xi_{1,t}+N_{4,f,t}\eta_{2,t}\rho_{2,t}\xi_{2,t}+N_{5,f,t}\eta_{3,t}\rho_{3,t}\xi_{3,t}\right)s_{1,f,t}\left(1-\alpha_{1,f,t+1}\right)\right)\\ N_{2,f,t+1}∼\text{Poisson}\left(\left(N_{2,f,t}\eta_{1,t}\rho_{1,t}\xi_{1,t}+N_{4,f,t}\eta_{2,t}\rho_{2,t}\xi_{2,t}+N_{5,f,t}\eta_{3,t}\rho_{3,t}\xi_{3,t}\right)s_{1,f,t}\alpha_{1,f,t+1}\right)\\ N_{3,f,t+1}∼\text{binomial}\left(N_{1,f,t},s_{2,f,t}\left(1-\alpha_{2,f,t+1}\right)\right)\\ N_{4,f,t+1}∼\text{binomial}\left(N_{1,f,t},s_{2,f,t}\alpha_{2,f,t}\right)+\text{binomial}\left(N_{2,f,t},s_{2,f,t}\right)\\ N_{5,f,t+1}∼\text{binomial}\left(N_{3,f,t}+N_{4,f,t}+N_{5,f,t},s_{3,f,t}\right) \end{array}.$$

For males we have similar expressions, but in addition we include immigrants:

$$\begin{array}{c} N_{1,m,t+1}∼\text{Poisson}\left(\left(N_{2,f,t}\eta_{1,t}\rho_{1,t}\left(1-\xi_{1,t}\right)+N_{4,f,t}\eta_{2,t}\rho_{2,t}\left(1-\xi_{2,t}\right)+N_{5,f,t}\eta_{3,t}\rho_{3,t}\left(1-\xi_{3,t}\right)\right)s_{1,m,t}\left(1-\alpha_{1,m,t+1}\right)\right)\\ N_{2,m,t+1}∼\text{Poisson}\left(\left(N_{2,f,t}\eta_{1,t}\rho_{1,t}\left(1‐\xi_{1,t}\right)+N_{4,f,t}\eta_{2,t}\rho_{2,t}\left(1‐\xi_{2,t}\right)+N_{5,f,t}\eta_{3,t}\rho_{3,t}\left(1‐\xi_{3,t}\right)\right)s_{1,m,t}\alpha_{1,m,t+1}\right)\\ N_{3,m,t+1}∼\text{binomial}\left(N_{1,m,t},s_{2,m,t}\left(1-\alpha_{2,m,t+1}\right)\right)\\ N_{4,m,t+1}∼\text{binomial}\left(N_{1,m,t},s_{2,m,t}\alpha_{2,m,t}\right)+\text{binomial}\left(N_{2,m,t},s_{2,m,t}\right)\\ N_{5,m,t+1}∼\text{binomial}\left(N_{3,m,t}+N_{4,m,t}+N_{5,m,t}+N_{6,m,t},s_{3,m,t}\right)\\ N_{6,m,t+1}∼\text{Poisson}\left(\omega_{t+1}\right) \end{array}.$$

*N*_*a,s,t*_ is the number of individuals in age class *a* of sex *s* (with levels *f* = female and *m* = male) in year *t*, *s*_*a*,*s*_ is annual age‐specific survival of sex *s* (*s*_1,*s*_: survival from fledging until the age of 1 year; *s*_2,*s*_: survival from the age of 1 year to the age of 2 years; *s*_3,*s*_: survival from the age of 2 years onwards), $\eta_{a}$ is breeding success, the age‐specific probability that a brood produced at least one fledgling ($\eta_{1}$: breeding success of 1‐year‐old females; $\eta_{2}$: breeding success of 2‐year‐old females; $\eta_{3}$: breeding success of 3‐year‐old or older females), $\rho_{a}$ is productivity, the age‐specific number of fledglings in a successful brood ($\rho_{1}$: number of fledglings raised by 1‐year‐old females; $\rho_{2}$: number of fledglings raised by 2‐year‐old females; $\rho_{3}$: number of fledglings raised by 3‐year‐old or older females), $\xi_{a}$ is the age‐specific proportion of females fledgling in a brood ($\xi_{1}$: fledgling sex ratio in broods raised by 1‐year‐old females; $\xi_{2}$: fledgling sex ratio in broods raised by 2‐year‐old females; $\xi_{3}$: fledgling sex ratio in broods raised by 3‐year‐old or older females), $\alpha_{a,s}$ is sex‐ and age‐specific probability of first reproduction ($\alpha_{1,s}$: probability that a 1‐year old individual of sex *s* reproduces; $\alpha_{2,s}$: probability that a 2‐year old individual of sex *s* reproduces; both are hidden parameters), and ω is the expected number of male immigrants (Table 1). We initially fitted a model that also allows female immigration. Because we found that female immigration was very low (see Appendix 3 in Supporting Information for details), we use a more parsimonious model where female immigration is assumed absent. Immigrants are assumed to reproduce in the year when they immigrate and to be 3‐years old or older, because only a minority of males is recruited before becoming 3‐years old.

**TABLE 1:** Definition of the symbols of the stage‐specific population sizes and of the demographic rates, used in the integrated population model.

Symbol	Definition
*N*_1,s,t_	Number of non‐breeding 1‐year old individuals of sex *s* in year *t*
*N*_2,s,t_	Number of breeding 1‐years old individuals of sex *s* in year *t*
*N*_3,s,t_	Number of non‐breeding 2‐years old individuals of sex *s* in year *t*
*N*_4,s,t_	Number of breeding 3‐years old individuals of sex *s* in year *t*
*N*_5,s,t_	Number of breeding at least 3‐years old individuals of sex *s* in year *t*
*N*_6,m,t_	Number of breeding at least 3‐years old male immigrants in year *t*
*s*_a,s,t_	Survival of a goshawk of sex *s* and age class *a* (*a* = {1, 2, 3}) from year *t* to year *t* + 1 (annual survival)
$\eta_{a,t}$	Probability that a brood of a female of age class *a* (*a* = {1, 2, 3}) in year *t* is successful (breeding success)
$\rho_{a,t}$	Number of nestlings fledged from a successful brood reared by a female of age class *a* (*a* = {1, 2, 3}) in year *t* (productivity)
$\xi_{a,t}$	Proportion of female fledglings in a brood reared by a female of age class *a* (*a* = {1, 2, 3}) in year *t* (fledgling sex ratio)
$\alpha_{a,s,t}$	Probability that an individual of sex *s* and age class *a* (*a* = {1, 2}) starts to reproduce in year *t* (recruitment)

We imposed temporal random effects on all parameters, that is, on all demographic rates and the recapture and dead recovery probabilities. Therefore, all of them have a time index (*t*), but this is omitted from the notation above for simplicity. For the parameter θ, we generally used $g\left(\theta_{t}\right)\sim \text{Normal}\left(\mu_{\theta },\sigma_{\theta }^{2}\right)$, where *g* is an appropriate link function (logit for probabilities, identity for number of fledglings, log for number of immigrants), $\mu_{\theta }$ is the long‐term mean of parameter θ on the chosen link scale, and $\sigma_{\theta }^{2}$ is the temporal variability of parameter θ on the chosen link scale.

The likelihood of the IPM is the jointly likelihood composed of the likelihoods of all seven data sets as shown in Appendix 2 in Supporting Information (Schaub & Kéry, 2022). We fitted the integrated population model using the Bayesian framework (Kéry & Schaub, 2012; Schaub & Kéry, 2022). We used the program NIMBLE (de Valpine et al., 2017, 2020) and specified vague priors for all parameters (Appendix 1, Table A‐1 in Supporting Information). We ran 3 Monte Carlo Markov chains (MCMC) for 110,000 iterations, discarding the first 10,000 as burn‐in and keeping every 50th value. This specification resulted in a MCMC sample size of 6000. We performed posterior‐predictive checks to assess the fit of the model (results presented in Appendix 1, Figure A‐2 in Supporting Information). NIMBLE code of the IPM is provided in Appendix S4 in Supporting Information, and R code for all analyses and data are available on the vogelwarte.ch Open Repository and Archive (Schaub et al., 2024). We generally report posterior means and the 95% credible intervals (CRI) in parentheses. For inference we also computed the probability that a particular parameter was larger than another parameter or differed from a fixed value (usually 0).

### 2.5 Prospective and retrospective population analyses

We examined the relationship between demographic rates and the population growth rates, that is, population dynamics, by applying prospective and retrospective perturbation analyses (Caswell, 2000). The temporal change in any demographic rate can have a direct, immediate effect on the population growth rate, or a delayed effect mediated by the change in population structure. First, we investigated how sensitive the population growth rate is to changes in demographic rates and population structure by calculating elasticities (Koons et al., 2017). Second, we performed a retrospective population analysis (Koons et al., 2016, 2017; Schaub & Kéry, 2022) to quantify how much the temporal changes in demographic rates and population structure have contributed to the change in the realized population growth rate.

Some of the temporal variation in demographic rates, and hence their effect on population growth, is due to environmental variation (changes in demographic rates that are caused by environmental factors and are the same for all individuals), and some is due to demographic stochasticity. We calculated the difference between the actual estimated population size and the expected population size obtained in the absence of demographic stochasticity, from which the population growth rates resulting from environmental stochasticity alone and from demographic stochasticity alone were calculated (Knape et al., 2023). The contribution of environmental stochasticity was calculated from the latter.

### 2.6 Testing for the evidence of density dependence

We assessed for evidence of density dependence at the population level by a simulation approach (Schaub & Kéry, 2022). This approach calculates the strength of density dependence as the regression coefficient of log population size for all but the first year against log population size for all but the last year (Dennis & Taper, 1994). We calculated the strength of density dependence from the IPM estimates and compared them with values obtained from a simulated population under exponential growth. The latter shows the expected relationship in the absence of density dependence, which is negative due to the non‐independence of the dependent (*N*_t_) and independent variables (*N*_t+1_). It is used as a null hypothesis against which the observed relationship is tested. If there was evidence of density dependence at the population level, we fitted further IPMs that included a link between population size and demographic rates: demographic parameters θ were modeled as $g\left(\theta_{t}\right)\sim \text{Normal}\left(\mu_{\theta }+\beta_{\theta }N_{t},\sigma_{\theta }^{2}\right)$, where $\beta_{\theta }$ is the strength of density dependence of parameter θ and *N*_*t*_ is the total number of females in year *t*. We used a model that included such relationships for all demographic parameters except male immigration, because modeling density dependence on immigration within an IPM requires knowledge of the possible mechanism (Schaub & Kéry, 2022), which we did not have. For each parameter, we then tested whether the 95% credible interval of the posterior distribution of the strength of density dependence included zero.

## 3 RESULTS

**FIGURE 1:**
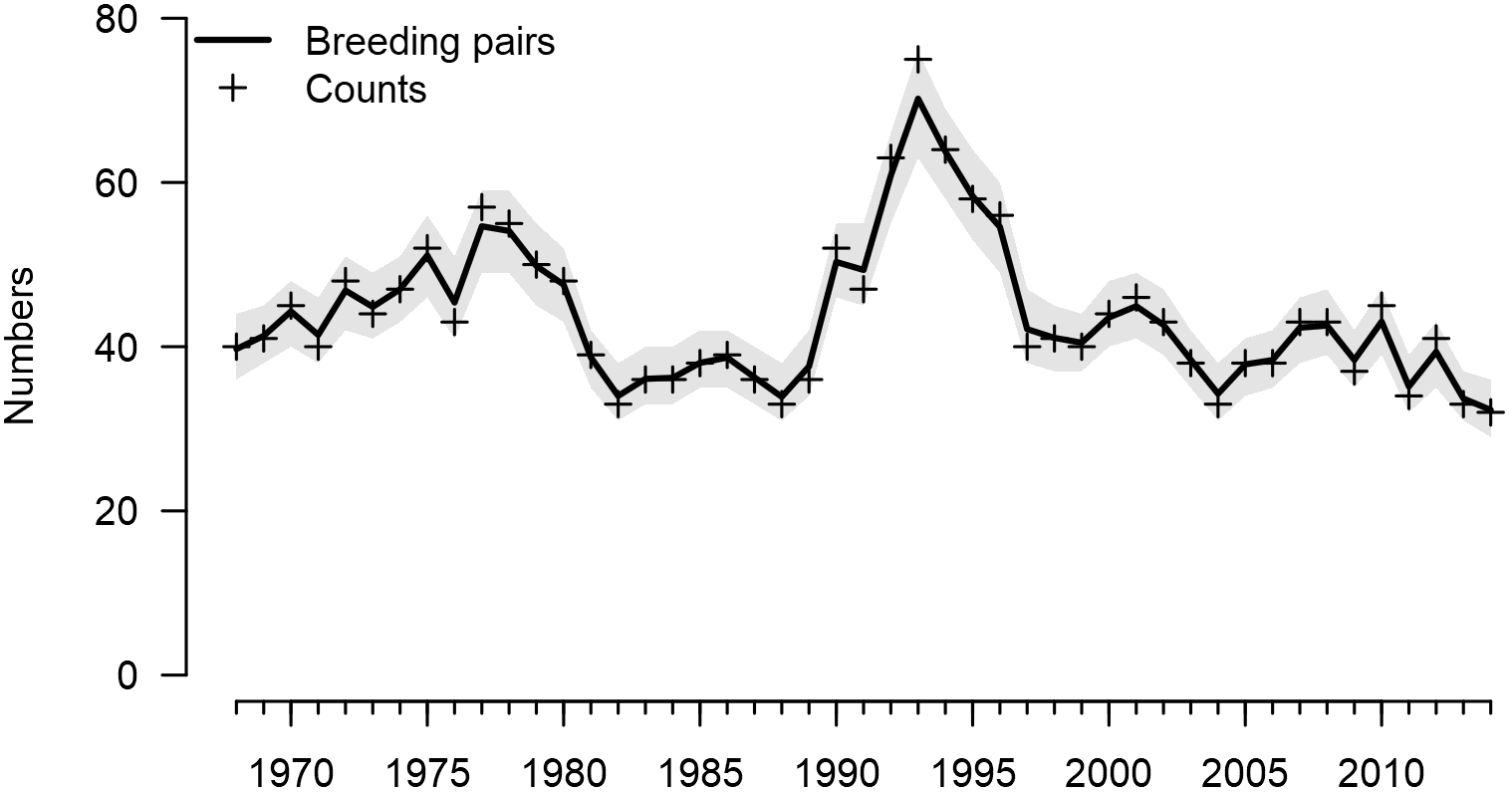
Number of goshawk breeding pairs in the study area from 1968 to 2014. Shown are the posterior means (solid line) and the 95% credible interval (gray layer) as obtained from the integrated population model, and the annual counts of the breeding pairs (+).

The integrated population model converged well and posterior predictive checks for component likelihoods were satisfactory (Appendix 1, Figure A‐2 in Supporting Information). The estimated number of breeding pairs fluctuated over the 47 years (Figure 1). They peaked in 1993 (71, CRI: 64–76) and was at its lowest in the last year of the study (32, CRI: 30–35). The coefficient of variation of the estimated number of breeding pairs was 19.4%. The geometric mean of the annual population growth rates was 0.994 (CRI: 0.989–0.999), indicating a slight decline of the population on average. The decline was 25% (CRI: 5%–41%) over the complete study period.

**FIGURE 2:**
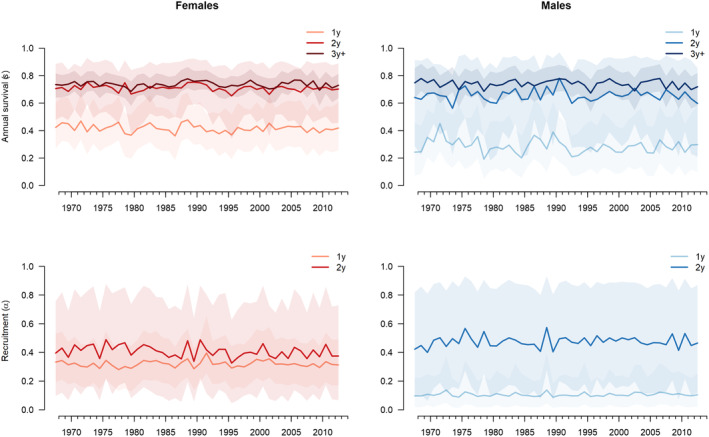
Estimated age‐specific annual survival of female and male goshawks (top row), and estimated age‐specific recruitment (i.e., probability of initiating reproduction). Solid lines show posterior means, the shaded areas show 95% credible intervals.

The sex‐ and age‐specific annual survival probabilities are shown in Figure 2 and the resighting and recovery probabilities in Appendix 1 (Figure A‐3 in Supporting Information). Survival fluctuated slightly over time but showed no long‐term trends. Mean survival increased with age for both sexes (Appendix 1, Table A‐2 in Supporting Information). First‐year survival of females was significantly higher than first‐year survival of males (*P*(*f* > *m*) = 0.99), whereas there was no evidence of a difference between sexes on second‐year and adult survival (*P*(*f* > *m*) = 0.68 and 0.39, respectively).

The probabilities of 1‐year old and 2‐years old individuals to start to reproduce (recruitment) were also stable over time with some fluctuations (Figure 2). While the probability of starting reproduction did not differ between age classes for females (*P*(1y < 2y) = 0.79), it was lower for 1‐year old males than for 2‐years old males (*P*(1y < 2y) = 1.00). Moreover, the probability of first‐time reproduction at the age of 1 year was lower in males than in females (*P*(*f* > *m*) = 1.00).

**FIGURE 3:**
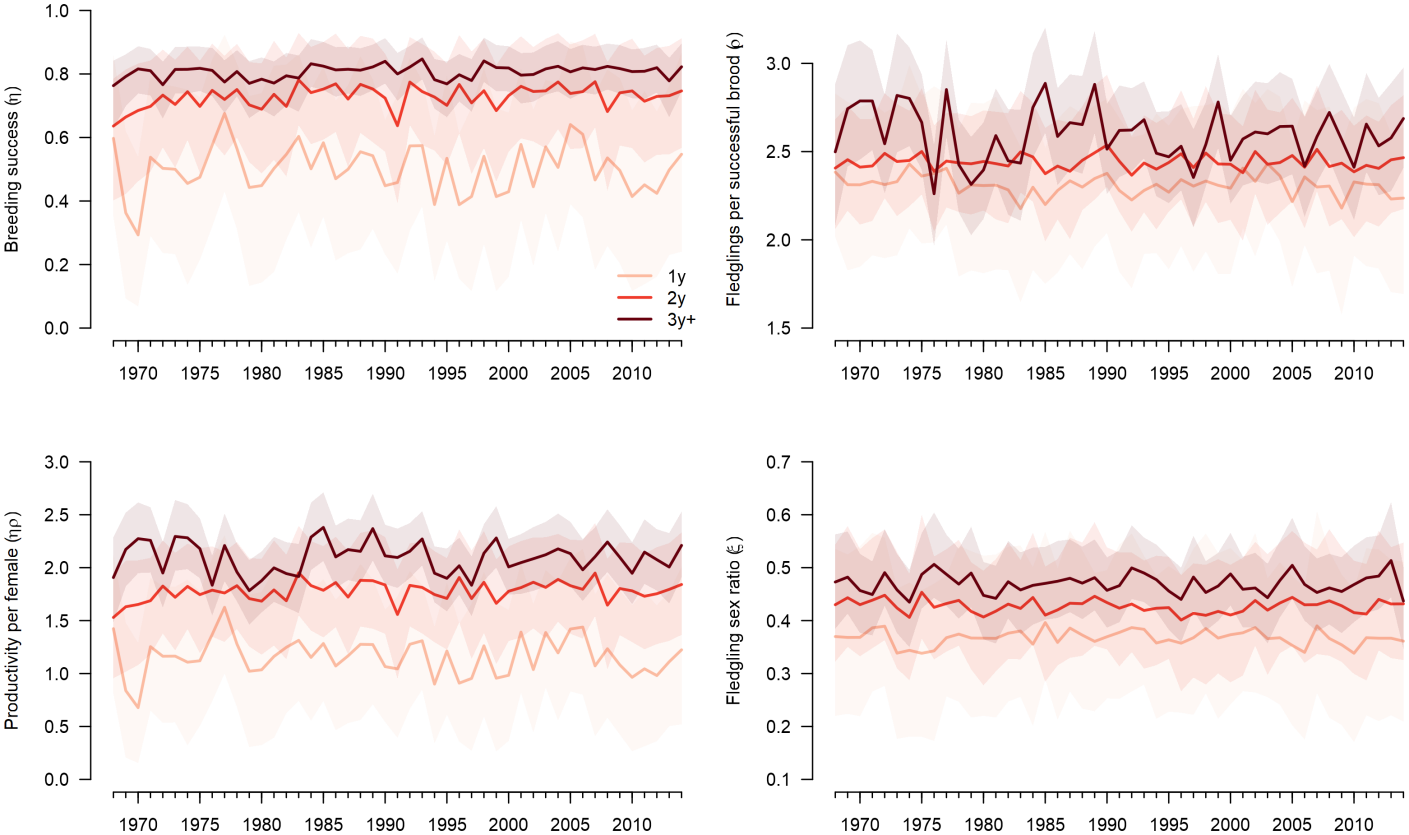
Estimated probability of successful reproduction, number of fledglings produced per successful brood, number of fledglings produced per initiated brood and fledgling sex ratio (proportion of females) produced by female goshawks of different age classes (1y: 1 year old; 2y: 2 years old; 3y+: at least 3 years old). Solid lines show posterior means, shaded areas show 95% credible intervals.

**FIGURE 4:**
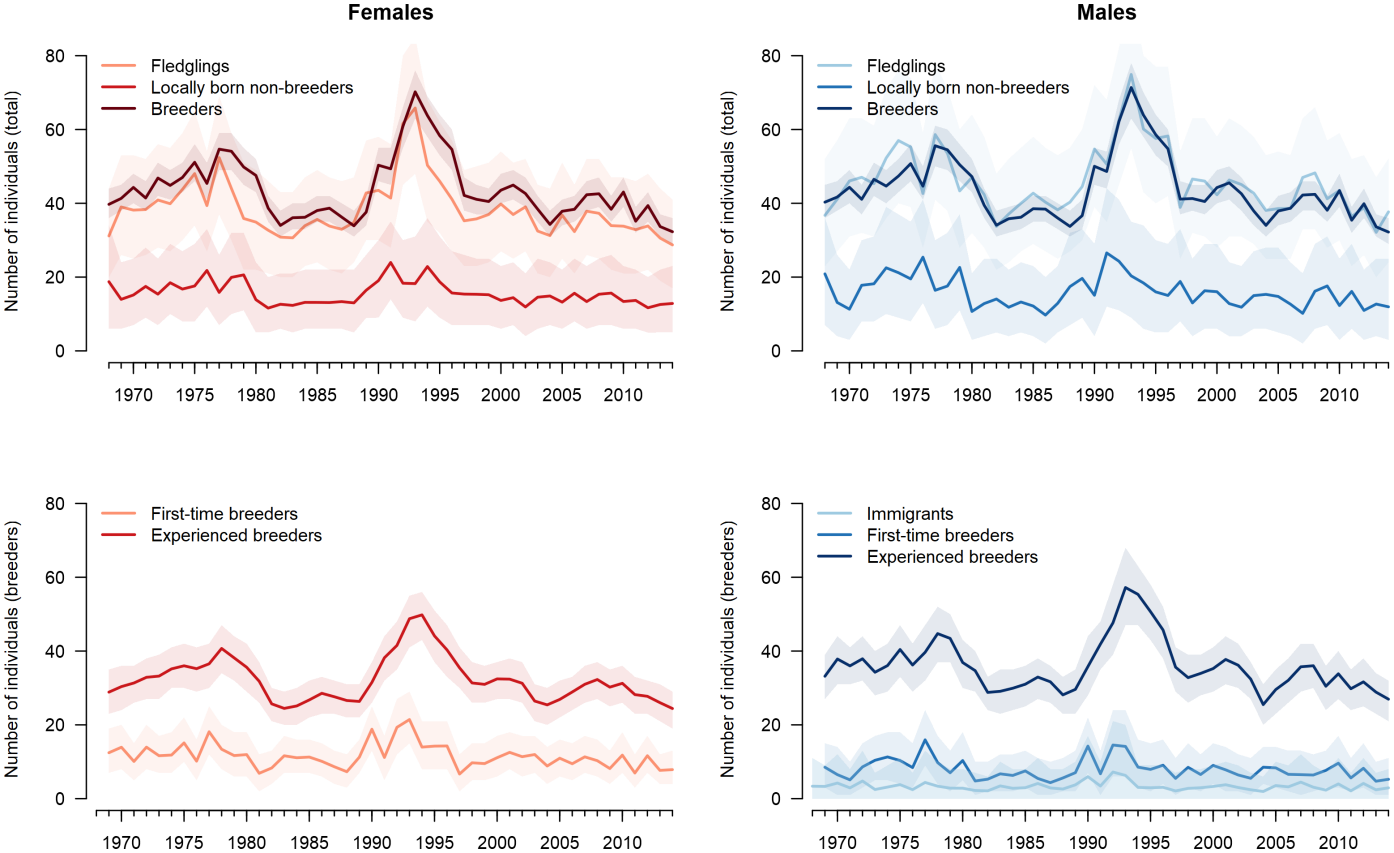
Composition and its changes over time of a goshawk population in Schleswig‐Holstein. The upper panels show the total number of females (left) and males (right) decomposed into fledglings produced, breeding individual and locally‐born non‐breeding individuals (floaters). The lower panels show a decomposition of the number of breeding females (left) and males (right) into locally born first‐time breeders and experienced breeders, for the males also the number of immigrants. The solid lines show the posterior means, the shaded areas the 95% credible intervals.

The productivity parameters changed with the age of the mother (Figure 3) and were variable over time. The breeding success and the number of fledglings reared per successful brood both increased with maternal age. Thus, the number of fledglings per initiated brood also increased with maternal age; it was 1.16 (CRI: 0.93–1.39), 1.80 (CRI: 1.61–1.98) and 2.10 (CRI: 2.01–2.19) fledglings per brood for 1‐year old, 2‐years old and at least 3‐years old females, respectively. The probability that the outcome was smaller in 1‐year than in 2‐years old females was 1.00 and that it was smaller in 2‐years than in at least 3‐years old females was 0.99. The fledgling sex ratio was male biased (Figure 3) in all maternal age classes, but it became less biased with increasing maternal age (Appendix 1, Table A‐2 in Supporting Information). The probability that the fledgling sex ratio of 1‐year old mothers was more male biased than that of 2‐years old mothers was 0.94, and the probability that the fledgling sex ratio differed between 2‐years old and at least 3‐years old mothers was 0.96. The average proportion of females among all fledglings produced was 0.46 (CRI: 0.33–0.58, Figure 4), and the annual proportions varied between years between 0.41 and 0.50.

At the end of a breeding season the goshawk population was composed of fledglings (mean proportion, both sexes combined: 0.41 [CRI: 0.33–0.49]), locally born non‐breeders (floaters, 0.16 [CRI: 0.08–0.24]) and breeders (0.43 [CRI: 0.37–0.50]) (if measured before breeding [fledglings excluded] the mean proportions were 0.27 floaters and 0.73 breeders), and these varied in concert over time (Figure 4). For males, the proportion of fledglings was slightly higher and the proportions of non‐breeders and breeders lower than for females. The breeding individuals can be decomposed in first‐time breeders, experienced breeders and immigrants. In females the average proportion of experienced breeders was 0.74 (CRI: 0.59–0.88), the remaining were first‐time breeders (0.26, CRI: 0.12–0.41). In males, the average proportion of experienced breeders was 0.74 (CRI: 0.56–0.90), the proportion of locally born first‐time breeders 0.18 (CRI: 0.05–0.35) and the proportion of immigrants 0.08 (CRI: 0.00–0.23). The sex ratio (proportion of males) of birds 1 year old or more was even (0.50, CRI: 0.43–0.57).

### 3.1 Prospective and retrospective analyses

**FIGURE 5:**
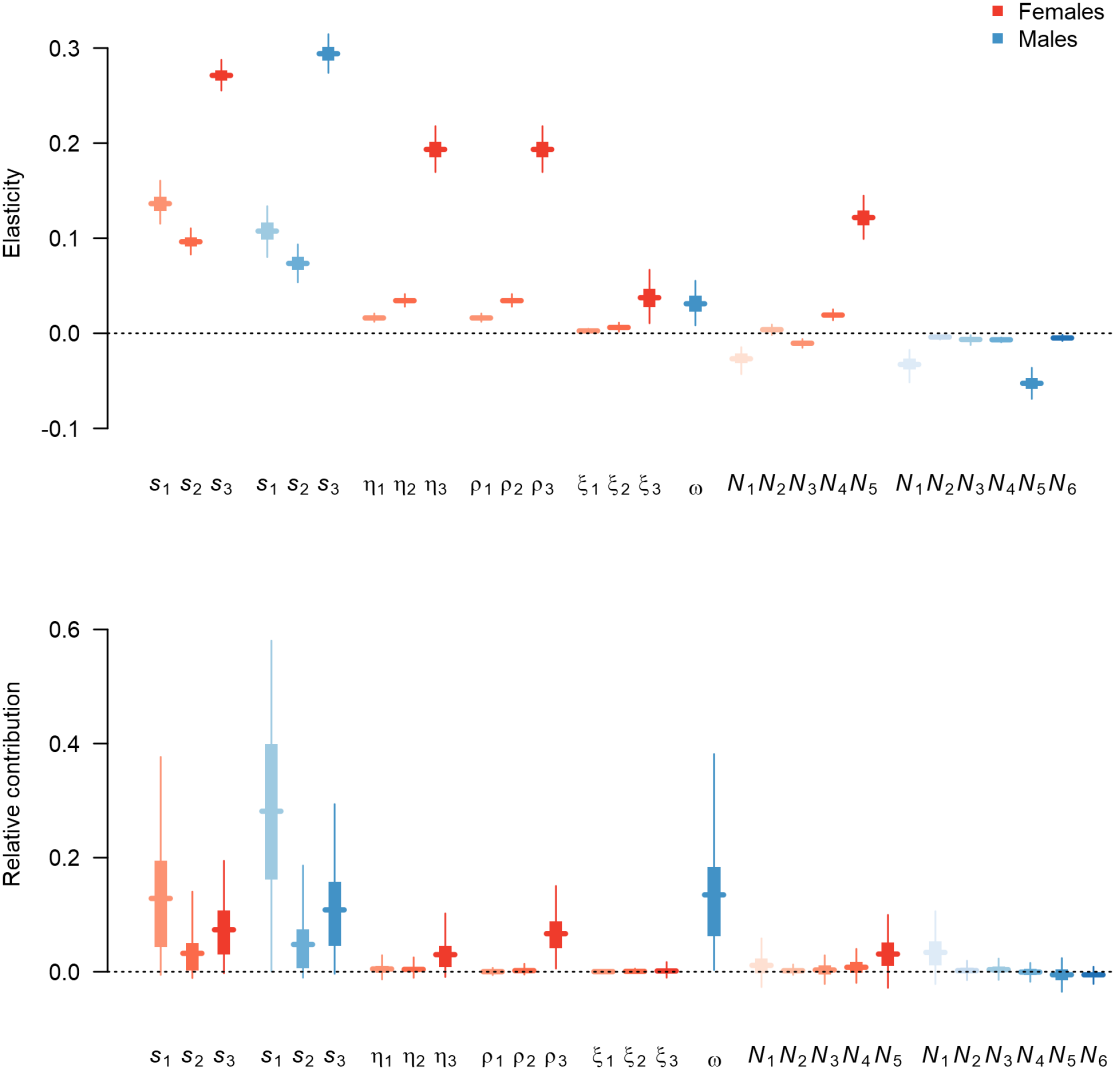
Sensitivities of demographic rates and of population structure to the realized population growth rate (expressed as elasticities, top) and the relative contributions of variation in the demographic components and in relative population structure to variation in the realized population growth rate (bottom). The horizontal lines show the posterior means, the vertical tick line the 50% credible intervals and the vertical thin lines the 95% credible intervals. *s*: Annual survival; η: Breeding success; ρ: Productivity; ξ: Fledgling sex ratio; ω: Male immigration; *N*: Relative population size; the subscripts refer to different age classes.

The realized population growth rate was more sensitive to changes in the demographic rates than to changes in the population stage structure (Figure 5, top panel). Among the demographic rates, the population was generally more sensitive to changes in the demographic rates of older than of younger individuals. The population was similarly sensitive to changes in female and male survival. It was equally sensitive to changes in breeding success and the number of fledglings per successful brood. Furthermore, there was little sensitivity to male immigration.

Increasing the proportion of breeding females would have a positive effect on the population growth, while increasing the proportion of non‐breeding females and males would have a negative effect on population growth. This is because an increasing proportion of adult males or non‐breeding females leads to a proportional decrease in breeding females, reducing the population growth rate.

Looking backwards in time, the variation in demographic rates has contributed more to the variation in the realized growth rate than the variation in population structure (Figure 5, bottom panel). In total, the summed relative contribution operating directly through demographic rates amounted to 0.86 (CRI: 0.74–0.94), the delayed contribution through changes in population structure to 0.14 (CRI: 0.06–0.26). Among the demographic rates, survival and immigration have contributed more than the productivity parameters. First‐year survival has contributed more than adult and second‐year survival, and on average male survival contributed slightly more than female survival due to higher temporal variation. Breeding success and productivity of adult females had contributed slightly to the variation in population growth rate, while these components of younger females contributed almost nothing. The variation in the sex ratio of fledglings had not contributed to population dynamics.

About half of the temporal contribution from demographic rates was due to environmental variation (0.49, CRI: 0.31–0.68), the other half was due to demographic stochasticity.

### 3.2 Assessment of density dependence

**FIGURE 6:**
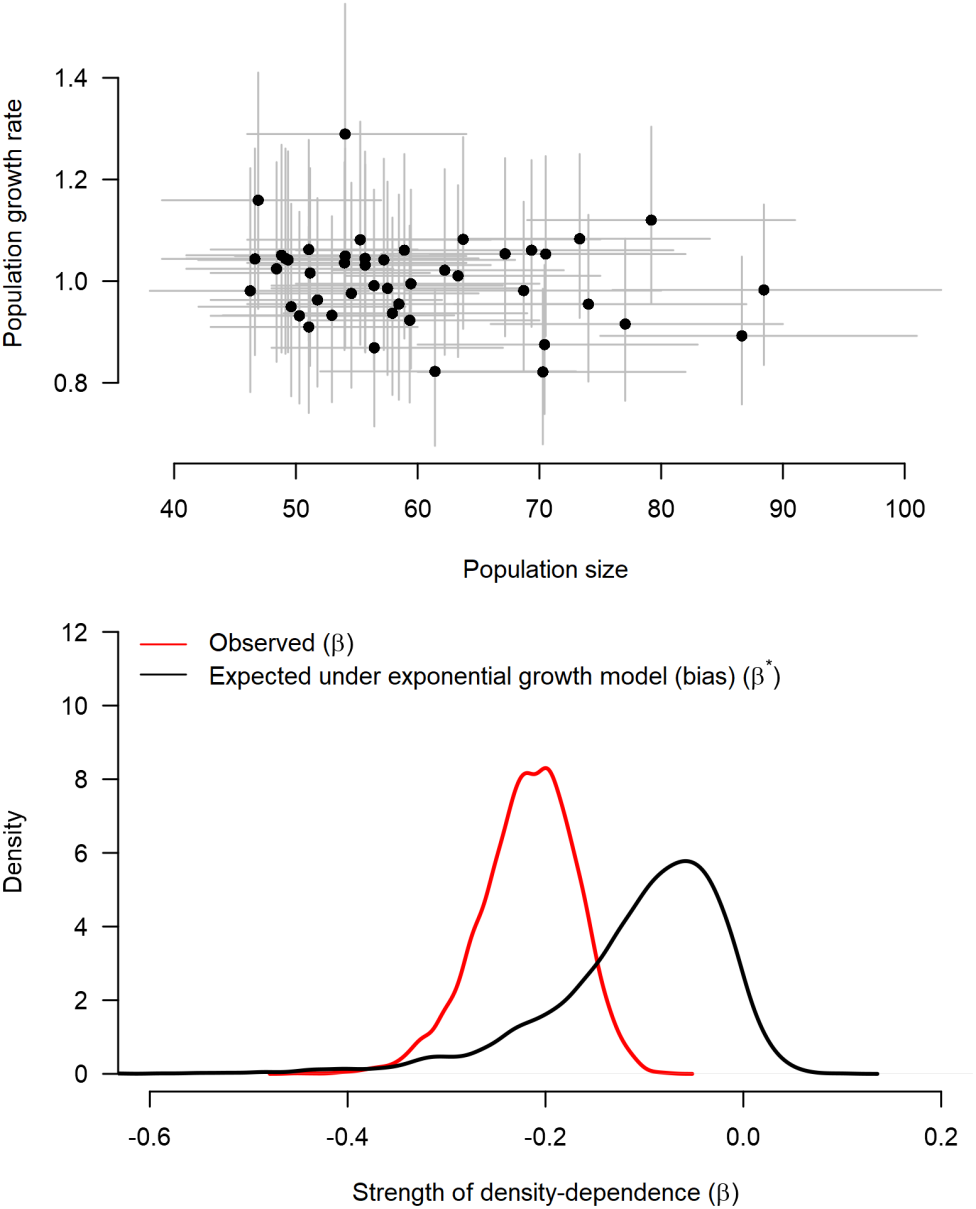
Scatterplot of population growth rate versus population size (top panel) and test of the strength of density dependence in a regression model (bottom panel). The dots show posterior means and the vertical and horizontal lines show 95% credible intervals. The lower panel shows the posterior distribution of the strength of density dependence for the actual data (red) and the reference distribution (black), generated in the absence of density dependence. The probability that the posterior distribution for the actual data is smaller than the reference distribution under *H*_0_, where there is no density dependence, is 0.87.

We used the total number of females to assess density dependence. The population growth rate decreased slightly with increasing population size (Figure 6, top panel), the regression coefficient of log population size against log population size in the previous year was −0.22 (CRI: −0.32, −0.13). However, the regression coefficient for a simulated population under exponential growth was also negative (mean: −0.11, CRI: −0.33, 0.02), and the probability that the former was smaller than the latter is 0.87 (Figure 6, bottom panel). Hence, there was some evidence of weak density dependence in the population.

**FIGURE 7:**
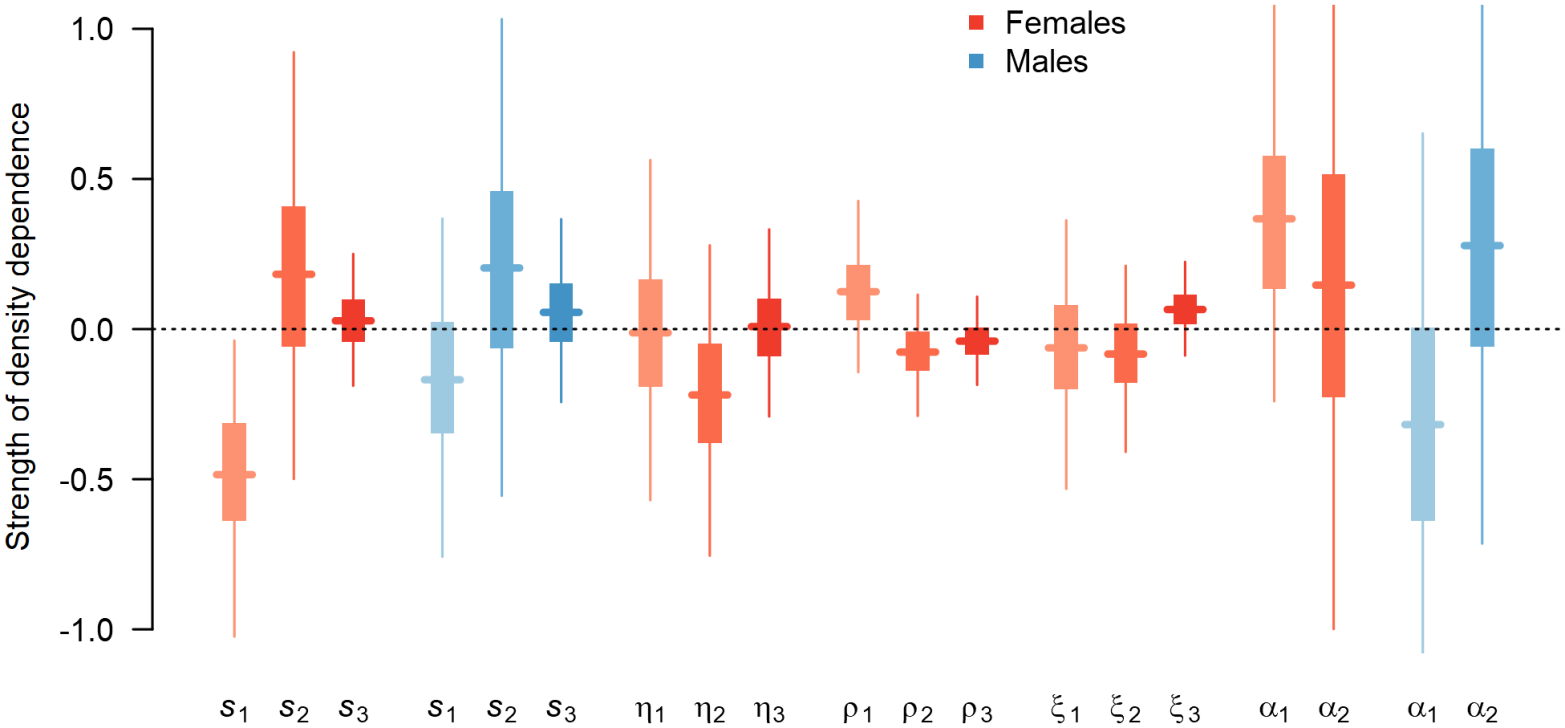
Strength of density dependence acting on the demographic rates. Estimates are the regression coefficients of the demographic rates against total female population size on the appropriate link scale. The horizontal lines show the posterior means, the vertical tick line the 50% credible intervals and the vertical thin lines the 95% credible intervals. *s*: Annual survival; η: Breeding success; ρ: Productivity; ξ: Fledgling sex ratio; the subscripts refer to different age classes.

The strength of density dependence operating on the demographic rates as obtained by fitting an IPM that included a link between demographic rates and total female population size is shown in Figure 7. With the exception of female first‐year survival, the 95% credible intervals of the posterior distributions of all parameters assessing the strength of density dependence acting on the demographic rates included zero. From an IPM that included density dependence on female first‐year survival only the strength of density dependence on that parameter was −0.168 (CRI: −0.400, 0.029).

## 4 DISCUSSION

The goshawk population studied for almost half a century, has declined slightly, with some ups and downs. About half of the variation in population growth rate was due to environmental variation in demographic rates, and the other half was due to demographic stochasticity. First‐year survival of both sexes, adult survival, and male immigration contributed most to the variation in population growth. The goshawk population produced more juvenile males than females, but due to the lower survival of males during the first year of life, fewer locally born males recruited to the population than females. These missing males were replaced by immigrants. Population growth was limited by weak density‐dependent regulation operating through first‐year female survival.

The estimated demographic rates generally compare well with those found in other goshawk populations. Mean survival probabilities are similar to those estimated for populations in Europe and North America (Kenward et al., 1999; Krüger, 2007; Reynolds et al., 2017; Ryttman, 1993; Tolvanen et al., 2017). The reproductive output of goshawks is strongly dependent on the age of the female (Krüger, 2005; Nielsen & Drachmann, 2003; Risch et al., 2004), but most studies on goshawk productivity have not taken maternal age into account and report average values between 1.5 and 2.1 nestlings per nest (Bezzel et al., 1997; Byholm, Ranta, et al., 2002; Kenward et al., 1999; Reynolds et al., 2017; Schlosser & Bühler, 2010). We used a parsimonious parameterization and distinguished between females aged 1, 2 and more than 2 years, so direct comparisons are not possible. Estimates from our population tend towards the upper range of reported values from other populations.

Goshawks are lazy dispersers; adult breeding site fidelity is very high (Tolvanen et al., 2017), and natal dispersal occurs over short distances (Byholm et al., 2003; Reynolds et al., 2017), with males dispersing over longer distances than females (Byholm et al., 2003). We found immigration of males, but not females, into our large study area, which is consistent with the finding that males move more than females. Finally, the estimated proportion of male fledglings of 0.54 is exactly the same as found in a meta‐analysis (Rutz, 2012).

### 4.1 Population dynamics

Goshawk populations often show long‐term stability, little fluctuations and strong density dependence (Rutz, Whittingham, & Newton, 2006). The main mechanisms for population regulation were the availability of territories (Krüger & Lindström, 2001) and food (Rutz & Bijlsma, 2006). Our population also showed long‐term stability despite the slight decline, but fluctuations were important and density dependence was rather weak. The underlying demographic reasons for population fluctuations were mainly stochastic effects, that is, temporal variation in demographic rates and demographic stochasticity, and only to a lesser extent density dependence.

Temporal variation in demographic rates leads to immediate changes in population growth and a delayed contribution through changes in population structure. Despite strong variation in some demographic rates, the stage and sex structure of the population varied little over time and contributed little to population dynamics. In contrast, about half of the temporal variation in population growth rate was due to direct effects of temporally varying demographic rates, mediated most strongly by first‐year survival of both sexes, followed by male immigration, adult survival of both sexes and marginally by adult female productivity. As with other long‐lived birds (Pfeiffer & Schaub, 2023; Sæther & Bakke, 2000), the goshawk population was most sensitive to changes in adult survival which explains why it contributed to changes in population growth rate despite its relatively low temporal variation (Figure 5). Goshawk population growth was much less sensitive to variation in first‐year survival, but this parameter contributed most to population dynamics due to high temporal variation.

We did not measure covariates that might explain temporal variation in demographic rates, but studies of goshawks have shown that spatial and temporal variability in food affects productivity (Byholm, Ranta, et al., 2002), nestling sex ratio (Byholm, Ranta, et al., 2002; Ranta et al., 2003; Rutz, 2012; Rutz & Bijlsma, 2006) and first‐year survival (Dewey & Kennedy, 2001; Ward & Kennedy, 1996; Wiens et al., 2006). Under conditions of high food availability, female nest attendance and nestling body mass are increased, both of which lead to higher fledgling success (Dewey & Kennedy, 2001; Tornberg et al., 2006). Large broods tend to result in male‐biased nestling sex ratios (Byholm, Ranta, et al., 2002). Despite a strong relationship between food supply and components of productivity, the population growth rate was little affected (this study and Krüger, 2007). This may be explained by the low sensitivity of goshawk population growth to changes in components of productivity.

As goshawks become independent from their parents, they enter a challenging period of their lives as they must be able to hunt on their own. The main cause of death for goshawks in their first year of life is starvation (Sunde, 2002; Tornberg et al., 2006; Wiens et al., 2006), suggesting that successful hunting is difficult, but skills improve with age (Rutz, Bijlsma, et al., 2006). Not surprisingly, food availability is the most important factor influencing first‐year survival (Wiens et al., 2006). Therefore, food availability appears to be an important driver of goshawk population dynamics that operates via first‐year survival (this study and Krüger, 2007). Interestingly, first‐year survival was also influenced by density dependent effects (this study and Krüger, 2007), which may also be related to direct competition for food. Food may also explain sex differences in first‐year survival. Because larger females are able to hunt larger prey than smaller males, they may take less risk in hunting, have better body condition than males (Sunde, 2002), and experience lower mortality in the first year than males (Kenward et al., 1999; Marcström & Kenward, 1981; Tornberg et al., 2006). We used dead recovery data to estimate first year survival, which therefore provides true survival unaffected by emigration from the study area (Burnham, 1993). However, spatial variation in recovery probability may still introduce some bias (Royle & Dubovsky, 2001). If the probability of recovery is greater in the study area than outside, and some males emigrate from the study area while females stay, our estimate of male survival will be negatively biased. It is therefore possible that the reported difference in first‐year survival between the sexes is actually smaller.

Demographic stochasticity, that is, stochastic variation in the realization of demographic processes, contributed about half of the variation in population size, even though the mean population size (males and females together) is more than 100 individuals, which is larger than the rule of thumb that demographic stochasticity becomes irrelevant. A possible reason for this could be structured variation between individuals, which is known to inflate demographic stochasticity if not accounted for (Kendall & Fox, 2002). We used three age classes and sex (for survival) to capture variation in demographic rates between individuals, but there is certainly more structured variation. We assumed that individuals at least 3 years old were the same, although there may be further age structure and senescence as shown in Krüger (2005). Moreover, breeding sites vary in quality (Krüger & Lindström, 2001) with consequences for productivity. Given the high site fidelity of breeders and a moderately long life span, this may lead to differences in productivity between individuals. Therefore, a population model with more structure (e.g., more age classes) needs to be used to more accurately assess the impact of demographic versus environmental stochasticity, but this was not possible with our data.

In addition to food, the availability of nesting sites is a major factor regulating goshawk populations (reviewed in Rutz, Whittingham, & Newton, 2006). The availability of nesting territories sets an upper limit for on population size for territorial species. At low population densities, the best territories should be occupied, and as density increases, more and more poor quality territories are occupied, potentially affecting population‐level demographic rates (Gill et al., 2001; Rodenhouse et al., 1997). Goshawks nest in forests, and the area of forests in our study area did not change during the study period, although some sites may have been temporarily unsuitable for nesting due to logging (Looft, 2017). Thus, while the upper limit of the potential territory did not change significantly, the non‐forested portion of the territory changed significantly due to agricultural intensification. Agricultural practices certainly affect the composition and density of breeding birds (Donald et al., 2001), but the food supply for goshawks may not have changed as they are generalist hunters. As territories become vacant and thus available for new birds to enter, they should be occupied by recruits, and demographically we would expect the probability of local recruitment or immigration to increase with declining population size. However, local recruitment and male immigration varied little over time, they contributed little to the variation in population growth rate and recruitment did not increase with declining population size. This suggests that the availability of nesting sites was not a major factor in the temporal fluctuations of the goshawk population.

### 4.2 Effects of sex

Most inferences from population dynamics studies are derived from female‐based population models (Lindström & Kokko, 1998; Miller & Inouye, 2011), where males are assumed to be an unlimited resource. However, if male and female dynamics differ, an understanding of population dynamics requires the inclusion of both sexes. We used a population model that explicitly included both sexes, but it appeared that sex was not very important, that is, we would have reached similar conclusions about the demographic drivers of the population without considering sex. There are two possible reasons for this. First, sex‐specific first‐year survival and male immigration compensate for the unequal sex ratio of juveniles, so that the sex ratio at maturity is equal. Adult survival differs little between sexes and the mating system of goshawks is monogamous. The dynamics of males and females are therefore similar (Figure 4) and we do not expect different results from female‐based models compared to two‐sex models. Second, sex may be important for goshawk population dynamics, but we did not find it because our two‐sexes population model did not include an explicit mating function (Miller & Inouye, 2011). The results of two‐sex population models with and without a mating function can differ (Gerber & White, 2014). However, during the development of our IPM, we tested a model that included a mating function. Most of the parameter estimates were very similar to those reported here, but the fit to the population counts was significantly less good. This was because male immigration is not identifiable in an IPM with a mating function (as it results in an excess of males). Although our inference from a two‐sex model without an explicit mating function is limited, we believe that the similarity of male and female adult dynamics is the main reason why we have not found a pronounced effect of sex on goshawk population dynamics.

The analysis of multiple data sets collected from the same population with integrated population models has proven to be powerful because it honestly leverages uncertainties among parameters and provides insights into the population structure (Plard et al., 2019; Schaub & Kéry, 2022). The models can be extended to two sexes with an explicit mating function (Tenan et al., 2016) or without it (e.g., Cleasby et al., 2017; Millsap et al., 2023; Rotelli et al., 2021; Schaub & Ullrich, 2021), allowing the impact of both sexes on the dynamics of a population to be assessed. To date, there is little empirical knowledge of how the variation in survival of each sex and sex ratio has contributed to population dynamics. Our study shows that the sexes differ only slightly in their contribution to population dynamics, but more empirical studies are needed for a more general understanding. Ideally, future empirical studies should use integrated population models as flexible and powerful tools to gain insight into the dynamics of highly age‐ and sex‐structured populations.

## AUTHOR CONTRIBUTIONS

**Michael Schaub:** Conceptualization (equal); formal analysis (lead); writing – original draft (lead). **Volkher Looft:** Data curation (lead); writing – review and editing (equal). **Floriane Plard:** Conceptualization (equal); formal analysis (supporting); writing – review and editing (equal). **Jan A. C. von Rönn:** Conceptualization (equal); formal analysis (supporting); writing – review and editing (equal).

## FUNDING INFORMATION

None.

## CONFLICT OF INTEREST STATEMENT

The authors declare that there are no conflicts of interest.

## Supporting information


Appendix S1.

## Data Availability

Data and code are available on the vogelwarte.ch Open Repository and Archive: https://doi.org/10.5281/zenodo.12565488 (Schaub et al., 2024).
