# Supplementary material for: Dynamics of a goshawk population across half a century is driven by the variation of first‐year survival
Source: Ecol Evol. 2024 Aug 1;14(8):e70058. doi: 10.1002/ece3.70058 (PMC11294034; doi:10.1002/ece3.70058)
Supplement: Supplementary file 1 — Appendix S1. [file ECE3-14-e70058-s001.pdf]

SUPPORTING INFORMATION

Dynamics of a goshawk population across half a century is driven by the variation of first-year survival

Michael Schaub[1], Volkher Looft[2], Floriane Plard[1,3], Jan A. C. von Rönn[1]

[1] Swiss Ornithological Institute, Seerose 1, 6204 Sempach, Switzerland

[2] Honigkamp 20, 24211 Postfeld, Germany

[3] Barraque de la Pinatelle, Tremoulet, 15500 Molompize, France

Corresponding author:

Michael Schaub; michael.schaub@vogelwarte.ch

Appendix 1

Additional figures and tables

Fig. A-1. Location of the study area (yellow box) in northern Germany (GER) just south of the border to Denmark (DK).

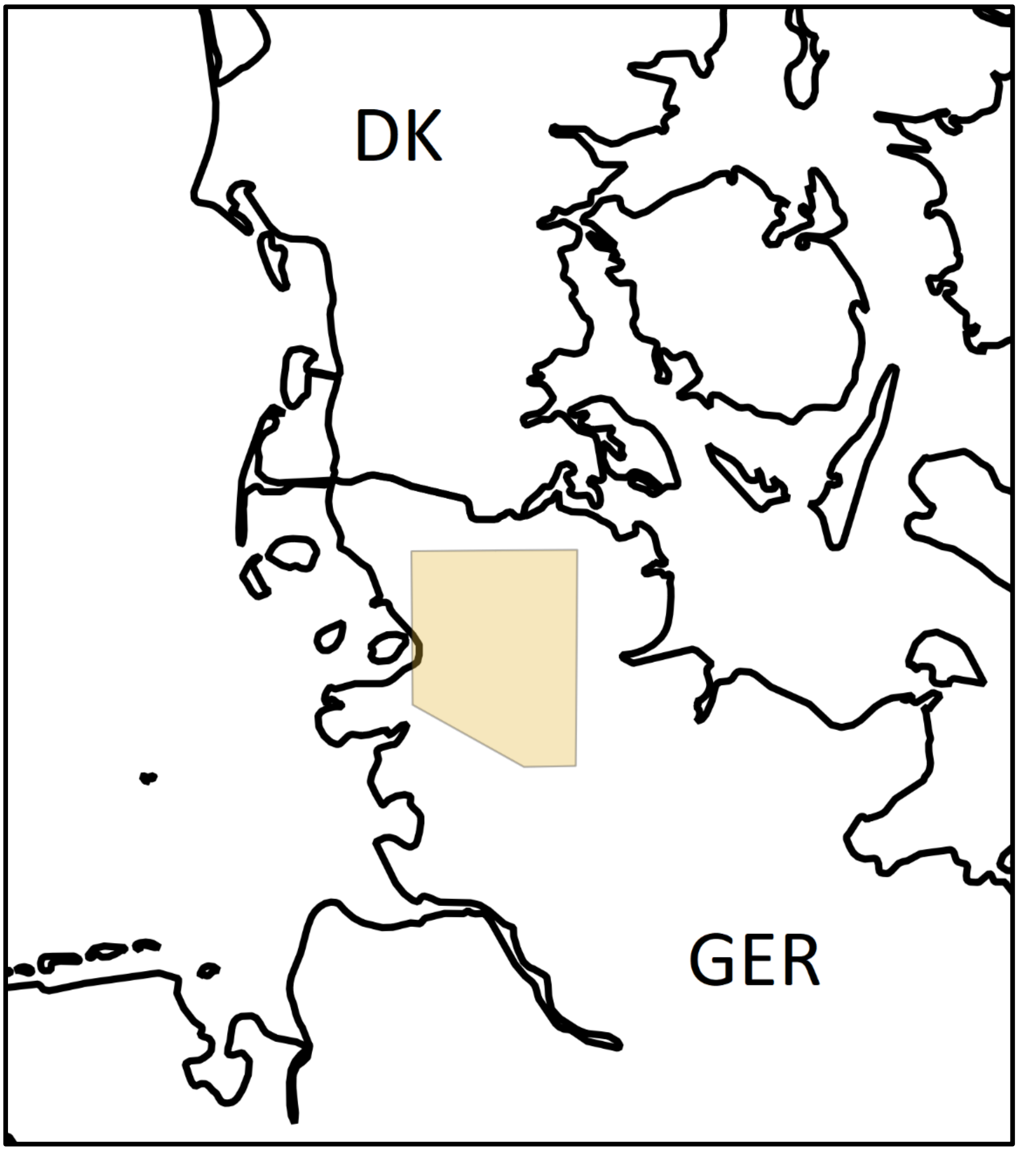

Fig. A-2. Posterior predictive checks of the main IPM (without density-dependence). As recommended by Schaub and Kéry (2022) we performed posterior-predictive checks for each data set included in the joint likelihood. We used the Freeman-Tukey test statistic as the discrepancy measure for the capture-recapture, the dead-recovery, the breeding success, the fledgling sex ratio and the breeding age ratio data, the Pearson χ^2 for number of fledglings and the mean absolute error for the population counts. The plots show the discrepancy measures of the observed data against the simulated data. Bayesian p-values (p_B) are also provided. The fits of all datasets appear satisfactory, except for the age ratio data, which are more variable than predicted by the model.

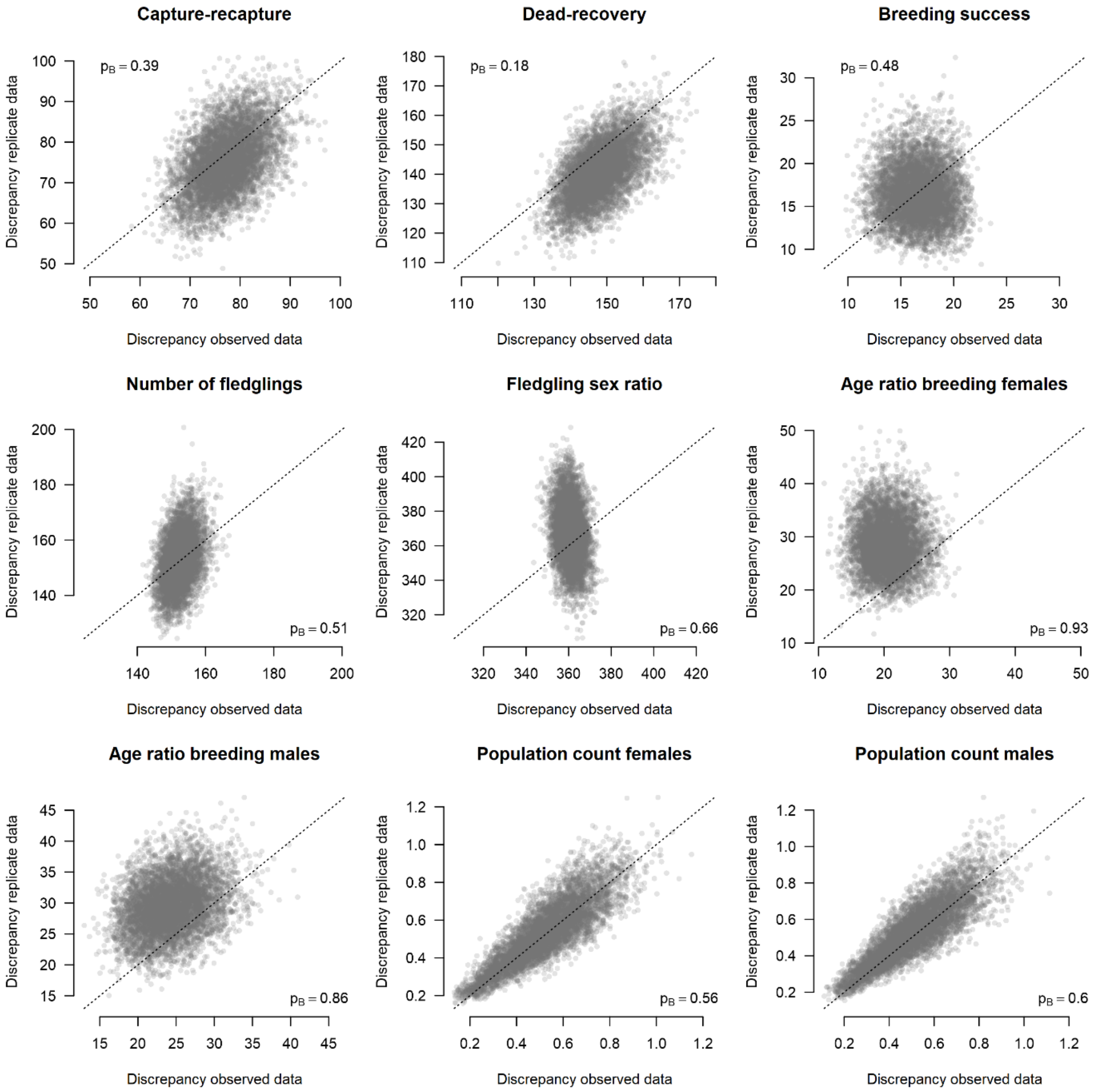

Fig. A-3. Estimated reencounter and ring-recovery probabilities. Shown are posterior means (fat lines) and the 95% credible intervals (vertical lines). The broken red lines and the red layer show the long-term means and associated 95% credible intervals. The encounter probabilities refer to the feather samples. There was a striking temporal pattern in the recovery of dead birds. In the first part it was generally higher and highly variable over time, whereas in the second part it was lower and fairly constant. This is partly due to the hunting of goshawks, which was legal until in the first part.

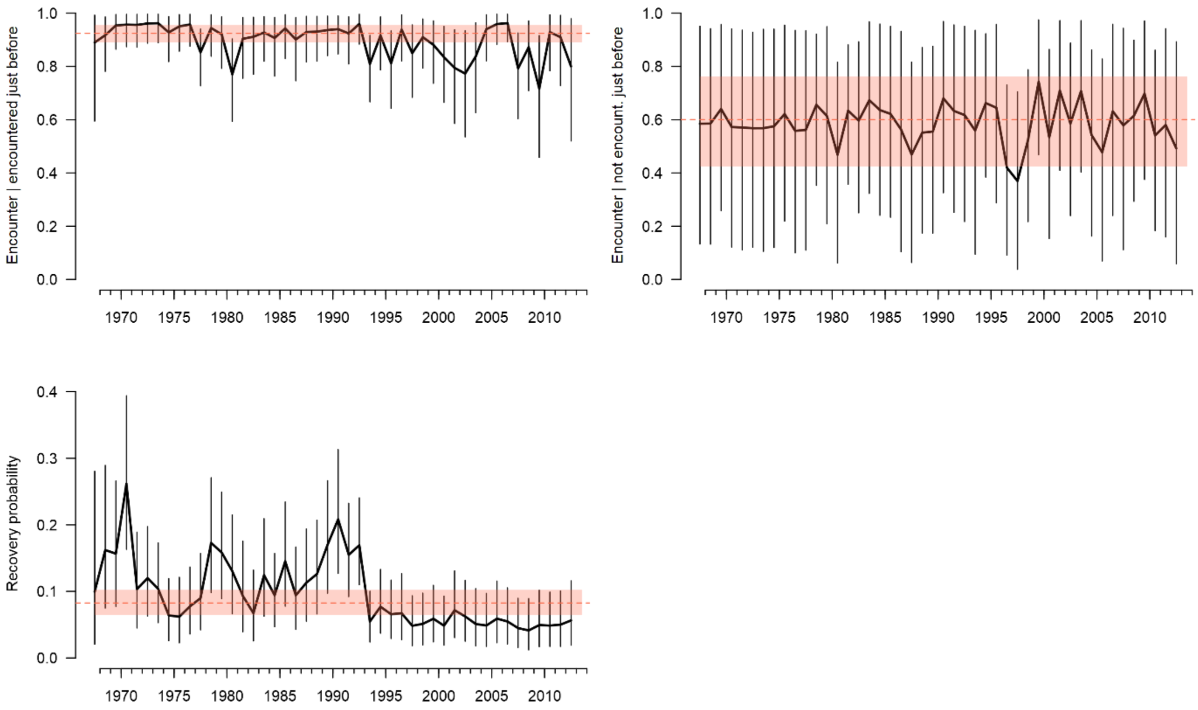

Table A-1. Prior distributions of all parameters in the integrated population model (without density-dependence).

Parameter	Significance	Prior distribution
$N_{1,s,1}$	Initial population size	discrete uniform (1, 28)
$N_{2,s,1}$	Initial population size	discrete uniform (1, 8)
$N_{3,s,1}$	Initial population size	discrete uniform (1, 12)
$N_{4,s,1}$	Initial population size	discrete uniform (1, 14)
$N_{5,s,1}$	Initial population size	discrete uniform (1, 44)
$\bar{s}_{a,s}$	mean survival	uniform (0, 1)
$\sigma_{s(a,s)}$	temporal SD of survival	uniform (0, 2)
$\bar{\eta}_a$	mean breeding success	uniform (0, 1)
$\sigma_{\eta(a)}$	temporal SD of breeding success	uniform (0, 2)
$\bar{\rho}_a$	mean productivity	normal (2, 100)
$\sigma_{\rho(a)}$	temporal SD of productivity	uniform (0, 2)
$\bar{\xi}_a$	mean nestling sex ratio	uniform (0, 1)
$\sigma_{\xi(a)}$	temporal SD of sex ratio	uniform (0, 2)
$\bar{\alpha}_a$	mean recruitment probability	uniform (0, 1)
$\sigma_{\alpha(a)}$	temporal SD of recruitment probability	uniform (0, 2)
$\bar{\omega}$	mean number of immigrants	uniform (0, 20)
σ_{ω}	temporal SD of immigrants	uniform (0, 2)
$\bar{p}_a$	mean resighting	uniform (0, 1)
σ_{ρ}	temporal SD of resighting	uniform (0, 3)
$\bar{r}$	mean recovery	uniform (0, 1)
σ_r	temporal SD of recovery	uniform (0, 3)
σ^2	Residual (observation) error	uniform (0.02, 0.3)

Table A-2. Estimates of the mean and the temporal variability of the demographic rates from the goshawk population in Schleswig 1968-2014 obtained from the integrated population model. The posterior means of the mean demographic rates are presented on the natural scale, the temporal variabilities are expressed as the temporal standard deviations on the appropriate link scales (logit for all probabilities, identity for the number of fledglings of successful broods). Given are also the limits of the 95% credible intervals (CRI).

Demographic parameter	Mean (CRI)	Temporal variability (CRI)
Annual first-year survival, females ($s_{1,f}$)	0.415 (0.347 – 0.494)	0.302 (0.061 – 0.665)
Annual second-year survival, females ($s_{2,f}$)	0.716 (0.607 – 0.819)	0.394 (0.023 – 1.023)
Annual adult survival, females ($s_{3,f}$)	0.739 (0.712 – 0.765)	0.217 (0.033 – 0.400)
Annual first-year survival, males ($s_{1,m}$)	0.275 (0.199 – 0.350)	0.517 (0.061 – 0.983)
Annual second-year survival, males ($s_{2,m}$)	0.671 (0.530 – 0.811)	0.622 (0.026 – 1.561)
Annual adult survival, males ($s_{3,m}$)	0.745 (0.706 – 0.785)	0.282 (0.055 – 0.536)
Breeding success of 1y females (η_1)	0.501 (0.410 – 0.590)	0.668 (0.148 – 1.266)
Breeding success of 2y females (η_1)	0.736 (0.671 – 0.801)	0.426 (0.053 – 0.918)
Breeding success of adult females (η_3)	0.809 (0.784 – 0.834)	0.267 (0.047 – 0.491)
Num. fledglings | success of 1y females (ρ_1)	2.311 (2.130 – 2.479)	0.180 (0.022 – 0.417)
Num. fledglings | success of 2y females (ρ_2)	2.439 (2.313 – 2.566)	0.119 (0.007 – 0.297)
Num. fledglings | success of adult females (ρ_3)	2.597 (2.516 – 2.677)	0.210 (0.132 – 0.299)
Fledgling sex ratio in broods of 1y females (ξ_1)	0.365 (0.302 – 0.433)	0.239 (0.031 – 0.682)
Fledgling sex ratio in broods of 2y females (ξ_2)	0.426 (0.381 – 0.470)	0.169 (0.008 – 0.452)
Fledgling sex ratio in broods of adult females (ξ_3)	0.470 (0.448 – 0.493)	0.149 (0.031 – 0.288)
Recruitment probability of 1y females ($\alpha_{1,f}$)	0.316 (0.242 – 0.403)	0.306 (0.028 – 0.721)
Recruitment probability of 2y females ($\alpha_{2,f}$)	0.399 (0.228 – 0.603)	0.634 (0.032 – 1.767)
Recruitment probability of 1y males ($\alpha_{1,m}$)	0.096 (0.047 – 0.160)	0.472 (0.017 – 1.432)
Recruitment probability of 2y males ($\alpha_{2,m}$)	0.473 (0.269 – 0.762)	0.653 (0.063 – 1.728)

Appendix 2

Description of the integrated population model

We defined a pre-breeding survey, two-sexes stochastic model with multiple stages for each sex. Stage classes 1 and 2 refer to individuals that are 1-year old and either breeding or non-breeding, respectively, stage classes 3 to 4 to 2-year-old individuals that are also either breeding or non-breeding, respectively, and stage class 5 refers to breeding individuals that are 3-year-old or older. Stage class 6 only occurs in males and includes immigrants (but see a model with additional female immigration in Appendix 3). We assume that all individuals 3-years old or older are breeding. The stage-specific numbers of individuals in year *t*+1 are functions of the numbers in year *t* and the demographic rates. To account for demographic stochasticity, we use Poisson and binomial distributions.

The stage-specific numbers of females in year *t*+1 is modeled as,

$$
\begin{aligned}
&N_{1,f,t+1} \sim Poisson\Big(\big(N_{2,f,t}\eta_{1,t}\rho_{1,t}\xi_{1,t} + N_{4,f,t}\eta_{2,t}\rho_{2,t}\xi_{2,t} + N_{5,f,t}\eta_{3,t}\rho_{3,t}\xi_{3,t}\big)s_{1,f,t}\left(1-\alpha_{1,f,t+1}\right)\Big)\\
&N_{2,f,t+1} \sim Poisson\Big(\big(N_{2,f,t}\eta_{1,t}\rho_{1,t}\xi_{1,t} + N_{4,f,t}\eta_{2,t}\rho_{2,t}\xi_{2,t} + N_{5,f,t}\eta_{3,t}\rho_{3,t}\xi_{3,t}\big)s_{1,f,t}\alpha_{1,f,t+1}\Big)\\
&N_{3,f,t+1} \sim binomial\Big(N_{1,f,t}, s_{2,f,t}\left(1-\alpha_{2,f,t+1}\right)\Big)\\
&N_{4,f,t+1} \sim binomial\left(N_{1,f,t}, s_{2,f,t}\alpha_{2,f,t}\right) + binomial\left(N_{2,f,t}, s_{2,f,t}\right)\\
&N_{5,f,t+1} \sim binomial\left(N_{3,f,t} + N_{4,f,t} + N_{5,f,t}, s_{3,f,t}\right)
\end{aligned}
.
$$

For males we have similar expressions, but in addition we include immigrants:

$$
\begin{aligned}
&N_{1,m,t+1} \sim Poisson\Big(\big(N_{2,f,t}\eta_{1,t}\rho_{1,t}\left(1-\xi_{1,t}\right) + N_{4,f,t}\eta_{2,t}\rho_{2,t}\left(1-\xi_{2,t}\right) + N_{5,f,t}\eta_{3,t}\rho_{3,t}\left(1-\xi_{3,t}\right)\big)s_{1,m,t}\left(1-\alpha_{1,m,t+1}\right)\Big)\\
&N_{2,m,t+1} \sim Poisson\Big(\big(N_{2,f,t}\eta_{1,t}\rho_{1,t}\left(1-\xi_{1,t}\right) + N_{4,f,t}\eta_{2,t}\rho_{2,t}\left(1-\xi_{2,t}\right) + N_{5,f,t}\eta_{3,t}\rho_{3,t}\left(1-\xi_{3,t}\right)\big)s_{1,m,t}\alpha_{1,m,t+1}\Big)\\
&N_{3,m,t+1} \sim binomial\Big(N_{1,m,t}, s_{2,m,t}\left(1-\alpha_{2,m,t+1}\right)\Big)\\
&N_{4,m,t+1} \sim binomial\left(N_{1,m,t}, s_{2,m,t}\alpha_{2,m,t}\right) + binomial\left(N_{2,m,t}, s_{2,m,t}\right)\\
&N_{5,m,t+1} \sim binomial\left(N_{3,m,t} + N_{4,m,t} + N_{5,m,t} + N_{6,m,t}, s_{3,m,t}\right)\\
&N_{6,m,t+1} \sim Poisson\left(\omega_{t+1}\right)
\end{aligned}
$$

$N_{a,s,t}$ is the number of individuals in age class *a* of sex *s* (with levels *f*=female and *m*=male) in year *t*, $s_{a,s}$ is annual age-specific survival of sex *s* ($s_{1,s}$: survival from fledging until the age of 1 year; $s_{2,s}$: survival from the age of 1 year to the age of 2 years; $s_{3,s}$: survival from the age of 2 years onwards), η_a is breeding success, the age-specific probability that a brood produced at least one fledgling (η_1 : breeding success of 1-year-old females; η_2 : breeding success of 2-

year-old females; η_3 : breeding success of 3-year-old or older females), ρ_a is productivity, the age-specific number of fledglings in a successful brood (ρ_1 : number of fledglings raised by 1-year-old females; ρ_2 : number of fledglings raised by 2-year-old females; ρ_3 : number of fledglings raised by 3-year-old or older females), ξ_a is the age-specific proportion of females fledgling in a brood (ξ_1 : fledgling sex ratio in broods raised by 1-year-old females; ξ_2 : fledgling sex ratio in broods raised by 2-year-old females; ξ_3 : fledgling sex ratio in broods raised by 3-year-old or older females), $\alpha_{a,s}$ is sex- and age-specific probability of first reproduction ($\alpha_{1,s}$: probability that a 1-year old individual of sex *s* reproduces; $\alpha_{2,s}$: probability that a 2-year old individual of sex *s* reproduces), and ω is the expected number of male immigrants. We initially fitted a model that also allows female immigration. Because we found that it was very low (see Appendix 3), we use a more parsimonious model where female immigration is assumed absent. Immigrants are assumed to reproduce in the year when they immigrate and to be 3-years old or older, because only a minority of males is recruited before becoming 3-years old. All demographic rates vary over time, i.e., are subject to environmental variation and, depending on the model fitted, on population size. Therefore, all of them have a time index (*t*), but this is omitted from the notation above for simplicity.

We next describe the likelihoods of all the sub-models used to analyse the seven data sets: 1) number of occupied territories (*n*=47 years), 2) sex- and age-specific (3 age classes) annual number of breeders (based on the collected feathers), 3) success (successful vs failed) of 1851 broods, 4) number of fledglings of successful broods (*n*=1334 broods), 5) fledgling sex ratio (*n*=1281 broods), 6) capture-recapture data based on the collected feathers from 462 adult individuals (341 females, 121 males), and 7) 319 (137 females, 182 males) dead-recoveries from a total of 3509 (1595 females, 1911 males) ringed nestlings.

The population count data (annual **number of occupied territories**) are analysed with a state-space model (de Valpine and Hastings, 2002), whose state process model is the stage-structured population model defined above. We use weakly informative prior distributions for the population sizes in the first year (see Appendix 1 Table A-1 for details). The observation model links the observed ($C_{s,t}$) with the estimated number of breeding individuals of sex *s* ($B_{s,t}$) using a log-normal distribution, $C_{s,t} \sim logNormal\left(B_{s,t}, \sigma^2\right)$. The

estimated numbers of breeding females and males in year t are $B_{f,t} = N_{2,f,t} + N_{4,f,t} + N_{5,f,t}$ and $B_{m,t} = N_{2,m,t} + N_{4,m,t} + N_{5,m,t} + N_{6,m,t}$, respectively, and σ^2 is the residual error of the IPM that includes observation errors and lack of fit.

The **sex and age-specific number of breeders** could be identified (1-year old, 2-years old, 3-years old or older) if feathers from them have been sampled under the nest. The observed numbers of 1-year old, 2-years old and older female breeders in year t (***k***) were modeled with multinomial distributions:

$$\boldsymbol{k}_{f,t,1:3} \sim multinomial\left(\left[\frac{N_{2,f,t}}{B_{f,t}}, \frac{N_{4,f,t}}{B_{f,t}}, \frac{N_{5,f,t}}{B_{f,t}}\right], \sum_{j=1}^{3} k_{f,t,j}\right).$$

For the males a similar expression was used, but the immigrants must be included which requires an assumption about their age. Since most locally born males are recruited at the age of 3 years, we also assume that immigrants are 3-years old. Hence, we have,

$$\boldsymbol{k}_{m,t,1:3} \sim multinomial\left(\left[\frac{N_{2,m,t}}{B_{m,t}}, \frac{N_{4,m,t}}{B_{m,t}}, \frac{N_{5,m,t} + N_{6,m,t}}{B_{m,t}}\right], \sum_{j=1}^{3} k_{m,t,j}\right).$$

The **success of monitored brood** was recorded, i.e., whether it failed or was successful and, if successful, how many fledglings were produced. The age class to which the breeding female belonged was usually known (n=1851 broods). The number of successful broods of females belonging to age class a in year t ($\eta_{a,t}$) was modelled with a binomial distribution as,

$$e_{a,t} \sim binomial\left(\eta_{a,t}, E_{a,t}\right),$$

where $E_{a,t}$ is the number of surveyed broods of females of age a in year t, and $\eta_{a,t}$ is the breeding success, as defined above.

The **number of fledglings of a successful brood** of female i belonging to age a in year t is modelled with a Normal distribution as,

$$f_{i(a),t} \sim Normal\left(\rho_{a,t}, \sigma_{\rho}^2\right),$$

where σ_{ρ}^2 is the variability of the number of fledglings, and $\rho_{a,t}$ the estimated number of fledglings (n=1334 broods). We used the Normal rather than the Poisson distribution because the latter was too overdispersed for the data at hand.

When nestlings are ringed, on average at the age of 20 days, their sex was identified based on their size (Bijlsma, 1994). The number of female chicks of brood i that was raised

by a female of age *a* in year *t* ($w_{i(a),t}$, **fledgling sex ratio**, was modeled with a binomial distribution as,

$$w_{i(a),t} \sim binomial\left(\xi_{a,t}, W_{i(a),t}\right),$$

where $W_{i(a),t}$ is the total number of fledglings of brood *i* and $\xi_{a,t}$ is the sex ratio as defined above (*n*=1281 broods).

Using individual identification based on feathers collected from breeding individuals, we constructed **capture histories** on an annual basis (*n*=1047; 752 females and 295 males). Goodness-of-fit tests performed with U-CARE (Choquet et al., 2009) suggested age effects on survival or the presence of transients. We do not believe that there were really transient or strong age effects in the survival of breeding birds, but that the inability to identify some individuals from feathers contributed to the lack of fit. In general, the probability of matching a feather to an individual increases with the number of feathers (number of years) that could be collected from that individual. Thus, individuals that survive for several years are more likely to be assigned the feather from the year the individual arrived than individuals that survived only a few years. This pattern results in apparent transient or age effects on survival, which bias survival estimates if not accounted for. We therefore removed the first record of all individuals, reducing the number of capture histories to 461 (341 females, 121 males). The resulting data therefore only included individuals that were at least two years old, and the estimated parameter was adult survival as defined above. Initial goodness-of-fit tests also indicated the presence of an immediate trap response (Pradel, 1993), i.e., the probability of encountering an individual in a given year depended on whether it was encountered in the previous year. We accounted for this effect by adapting the model for the reencounter probabilities (Pradel, 1993). We constructed sex-specific m-arrays ($\boldsymbol{m}_s$) from the individual capture histories (Williams et al., 2002) and analysed them using multinomial distributions,

$$\boldsymbol{m}_{s,t,1:T} \sim multinomial\left(\boldsymbol{\pi}_{s,t,1:T}, \sum_{j=1}^{T} m_{s,t,j}\right),$$

where *T* is the number of years and $\boldsymbol{\pi}_s$ is a matrix whose elements are functions of sex-specific adult survival ($s_{3,s,t}$) and of two recapture probabilities that are defined as the probability of reencountering an individual in year *t* if it was either encountered in *t*-1 ($p_{1,t}$) or not encountered in *t*-1 ($p_{2,t}$).

The **dead-recovery data** originating from nestlings marked in the study that were eventually found dead and whose ring was reported. A total of 3506 nestlings (1595 females, 1911 males) were ringed of which a total of 319 (137 females, 182 males) were found dead. The vast majority of recoveries originated from the study area (Looft, 2017). We transformed the individual capture-histories into a sex-specific dead-recovery m-array ($\boldsymbol{d}_s$) (Williams et al., 2002) and used the multinomial distribution,

$$\boldsymbol{d}_{s,t,1:T} \sim multinomial\left(\varpi_{s,t,1:T}, \sum_{j=1}^{T} d_{s,t,j} \right),$$

where T is the number of years, and ϖ_s is a function of sex and age-dependent survival probabilities ($s_{1,s,t}$, $s_{2,s,t}$, and $s_{3,s,t}$, as defined above) and of the probabilities to report a dead individual (r_t, recovery probability).

The likelihood of the IPM is the jointly likelihood composed of the likelihoods of all seven data sets as introduced above (Schaub and Kéry, 2022). We fitted the integrated population model using the Bayesian framework (Kéry and Schaub, 2012; Schaub and Kéry, 2022). We used the program NIMBLE (de Valpine et al., 2017; de Valpine et al., 2020) and specified vague priors for all parameters. We ran 3 Monte Carlo Markov chains (MCMC) for 110,000 iterations, discarding the first 10,000 as burn-in and keeping every 50th value. This specification resulted in a MCMC sample size of 6,000. The IPM provided estimates of all the parameters defined above, i.e. annual stage-structured population sizes, demographic rates and nuisance parameters. We performed posterior-predictive checks to assess the fit of the model (results presented in Appendix 1 Fig. A-2). NIMBLE code for the IPM is provided in Appendix 4, R code for all analyses and data are available on the vogelwarte.ch Open Repository and Archive: https://doi.org/10.5281/zenodo.12565488.

Appendix 3

Assessment of female immigration

To assess how much female immigration has occurred in the goshawk population, we fitted a structurally very similar IPM to the one used in the main part of the paper. The only difference was that it also allowed for female immigration. Therefore, for females, we added a 6th state as $N_{6,f,t+1} \sim Poisson(\omega_{f,t+1})$, where $\omega_{f,t+1}$ is the expected number of female immigrants in year *t*+1. We specified a uniform prior U(0, 20) for the expected number of immigrants, and the model also needed few additional changes (e.g. the observation model needs to include the immigrants as well, and female immigrants contribute to reproduction). The NIMBLE code for this model is available on the vogelwarte.ch Open Repository and Archive: https://doi.org/10.5281/zenodo.12565488.

Estimates of the mean number of female immigrants are shown in the figure below. For comparison, we also show the estimated number of male immigrants from the same model and from the main IPM, which only allowed male immigration. The figure shows the ranges of the 95% and 50% credible intervals and the median (thick horizontal line). Female immigration was significantly lower than male immigration, and male immigration from both models was very similar, suggesting that whether or not female immigrants were considered did not strongly affect the estimates of male immigration, nor the estimates of other demographic rates (not shown). Note that the prior for immigration had support for positive values only, that is why the estimated number of immigrants is positive and different from zero.

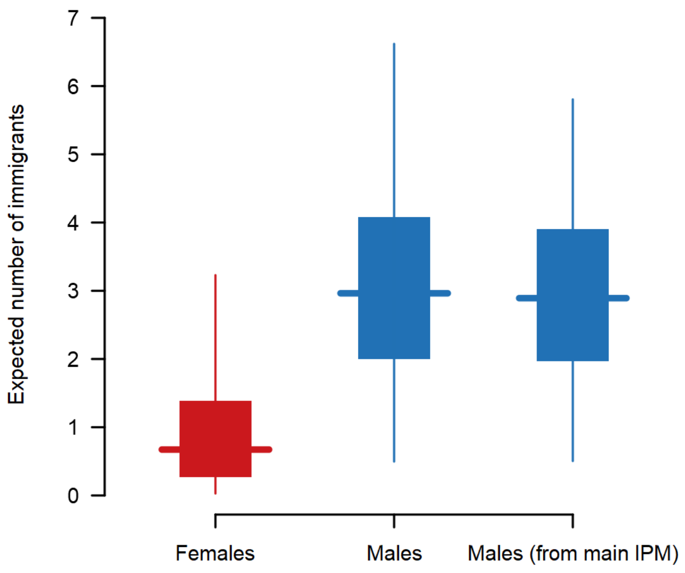

Appendix 4

NIMBLE code for the integrated population model

```
code_ipm1 <- nimbleCode({
# 1. Priors and linear models
# 1.1. CMR data
for (t in 1:(nyears-1)){
  for (k in 1:2){
    phi[k,t] <- s[3,k,t]
    for (a in 1:2){
      p[a,k,t] <- ilogit(lp[a,t])
    } #a

# 1.2. Dead recovery data
    for (a in 1:3){
      ls[a,k,t] ~ dnorm(mean.ls[a,k], sd=sigma.s[a,k])
      s[a,k,t] <- ilogit(ls[a,k,t])
    } #a
    r[k,t] <- ilogit(lr[t])
  } #k
  for (a in 1:2){
    lp[a,t] ~ dnorm(mean.lp[a], sd=sigma.p[a])
  } #a
  lr[t] ~ dnorm(mean.lr, sd=sigma.r)
} #t
for (k in 1:2){
  for (a in 1:3){
    mean.s[a,k] ~ dunif(0, 1)
    mean.ls[a,k] <- logit(mean.s[a,k])
    sigma.s[a,k] ~ dunif(0, 2)
  } #a
} #k
for (a in 1:2){
  mean.p[a] ~ dunif(0, 1)
  mean.lp[a] <- logit(mean.p[a])
  sigma.p[a] ~ dunif(0, 3)
}
mean.r ~ dunif(0, 1)
mean.lr <- logit(mean.r)
sigma.r ~ dunif(0, 3)

# 1.3. Probability of breeding success
for (a in 1:3){
  for (t in 1:nyears){
    leta[a,t] ~ dnorm(mean.leta[a], sd=sigma.eta[a])
  } #t
  eta[a,1:nyears] <- ilogit(leta[a,1:nyears])
  mean.eta[a] ~ dunif(0, 1)
  mean.leta[a] <- logit(mean.eta[a])
  sigma.eta[a] ~ dunif(0, 2)
} #a

# 1.4. Number of chicks given success
for (a in 1:3){
  for (t in 1:nyears){
    rho[a,t] ~ dnorm(mean.rho[a], sd=sigma.rho[a])
  } #t
  mean.rho[a] ~ dnorm(2, 0.01)
  sigma.rho[a] ~ dunif(0, 2)
```

```
} #a
sigma.chicks ~ dunif(0, 2)

# 1.5. Sex ratio of chicks
for (a in 1:3){
  for (t in 1:nyears){
    lxi[a,t] ~ dnorm(mean.lxi[a], sd=sigma.xi[a])
  } #t
  xi[a,1:nyears] <- ilogit(lxi[a,1:nyears])
  mean.xi[a] ~ dunif(0, 1)
  mean.lxi[a] <- logit(mean.xi[a])
  sigma.xi[a] ~ dunif(0, 2)
} #a

# 1.6. Recruitment probability (hidden parameter)
for (k in 1:2){
  for (a in 1:2){
    for (t in 1:(nyears-1)){
      lalpha[a,k,t] ~ dnorm(mean.lalpha[a,k], sd=sigma.alpha[a,k])
      alpha[a,k,t] <- ilogit(lalpha[a,k,t])
    } #t
    mean.alpha[a,k] ~ dunif(0, 1)
    mean.lalpha[a,k] <- logit(mean.alpha[a,k])
    sigma.alpha[a,k] ~ dunif(0, 2)
  } #a
} #k

# 1.7. Male immigration
for (t in 1:nyears){
  log.omega[t] ~ dnorm(mean.lomega, sd=sigma.omega)
  omega[t] <- exp(log.omega[t])
}
sigma.omega ~ dunif(0, 2)
mean.omega ~ dunif(0, 20)
mean.lomega <- log(mean.omega)

# 1.8. Residual / observation error
sigma.obs ~ dunif(0.02, 0.3)

# 1.9. Priors for the initial population size: discrete uniform
distributions
for (a in 1:5){
  N[a,1,1] ~ dcat(pNinit[a,])
  N[a,2,1] ~ dcat(pNinit[a,])
}
I[1] ~ dpois(omega[1])


# 2. Likelihoods
# 2.1 State-space model for population counts
# Process model of the state-space model: our model of population dynamics
for (t in 1:(nyears-1)){
  # Females
  F[1,t] ~ dpois(N[2,1,t] * eta[1,t] * rho[1,t] * xi[1,t])              #
Total number of female fledglings produced by 1y
  F[2,t] ~ dpois(N[4,1,t] * eta[2,t] * rho[2,t] * xi[2,t])              #
Total number of female fledglings produced by 2y
  F[3,t] ~ dpois(N[5,1,t] * eta[3,t] * rho[3,t] * xi[3,t])              #
Total number of female fledglings produced by adults

  N[1,1,t+1] ~ dbin(s[1,1,t] * (1-alpha[1,1,t]), (F[1,t] + F[2,t] +
F[3,t]))   # 1y NB
```

```
  N[2,1,t+1] ~ dbin(s[1,1,t] * alpha[1,1,t], (F[1,t] + F[2,t] + F[3,t]))
# 1y B

  N[3,1,t+1] ~ dbin(s[2,1,t] * (1-alpha[2,1,t]), N[1,1,t])
  n[1,1,t] ~ dbin(s[2,1,t] * alpha[2,1,t], N[1,1,t])
  n[2,1,t] ~ dbin(s[2,1,t], N[2,1,t])

  N[4,1,t+1] <- n[1,1,t] + n[2,1,t]
  N[5,1,t+1] ~ dbin(s[3,1,t], (N[3,1,t] + N[4,1,t] + N[5,1,t]))

  # First-time breeders of different ages
  FB[1,1,t] <- N[2,1,t+1]
  FB[2,1,t] <- n[1,1,t]
  FB[3,1,t] ~ dbin(s[3,1,t], N[3,1,t])

  # Males
  M[1,t] ~ dpois(N[2,1,t] * eta[1,t] * rho[1,t] * (1-xi[1,t]))            #
Total number of male fledglings produced by 1y
  M[2,t] ~ dpois(N[4,1,t] * eta[2,t] * rho[2,t] * (1-xi[2,t]))            #
Total number of male fledglings produced by 2y
  M[3,t] ~ dpois(N[5,1,t] * eta[3,t] * rho[3,t] * (1-xi[3,t]))            #
Total number of male fledglings produced by adults

  N[1,2,t+1] ~ dbin(s[1,2,t] * (1-alpha[1,2,t]), (M[1,t] + M[2,t] +
M[3,t]))   # 1y NB
  N[2,2,t+1] ~ dbin(s[1,2,t] * alpha[1,2,t], (M[1,t] + M[2,t] + M[3,t]))
# 1y B

  N[3,2,t+1] ~ dbin(s[2,2,t] * (1-alpha[2,2,t]), N[1,2,t])
  n[1,2,t] ~ dbin(s[2,2,t] * alpha[2,2,t], N[1,2,t])
  n[2,2,t] ~ dbin(s[2,2,t], N[2,2,t])

  N[4,2,t+1]  <- n[1,2,t] + n[2,2,t]
  N[5,2,t+1] ~ dbin(s[3,2,t], (N[3,2,t] + N[4,2,t] + N[5,2,t] + I[t]))
  I[t+1] ~ dpois(omega[t+1])

  # First-time breeders of different ages
  FB[1,2,t] <- N[2,2,t+1]
  FB[2,2,t] <- n[1,2,t]
  FB[3,2,t] ~ dbin(s[3,2,t], N[3,2,t])
}

# Number of fledglings produced in the last study year
F[1,nyears] ~ dpois(N[2,1,nyears] * eta[1,nyears] * rho[1,nyears] *
xi[1,nyears])
F[2,nyears] ~ dpois(N[4,1,nyears] * eta[2,nyears] * rho[2,nyears] *
xi[2,nyears])
F[3,nyears] ~ dpois(N[5,1,nyears] * eta[3,nyears] * rho[3,nyears] *
xi[3,nyears])
M[1,nyears] ~ dpois(N[2,1,nyears] * eta[1,nyears] * rho[1,nyears] * (1-
xi[1,nyears]))
M[2,nyears] ~ dpois(N[4,1,nyears] * eta[2,nyears] * rho[2,nyears] * (1-
xi[2,nyears]))
M[3,nyears] ~ dpois(N[5,1,nyears] * eta[3,nyears] * rho[3,nyears] * (1-
xi[3,nyears]))

# Observation model of the state-space model
for (t in 1:nyears){
  lcountf[t] ~ dnorm(log(N[2,1,t] + N[4,1,t] + N[5,1,t]), sd=sigma.obs)
  lcountm[t] ~ dnorm(log(N[2,2,t] + N[4,2,t] + N[5,2,t] + I[t]),
sd=sigma.obs)
}
```

```
# 2.2. Observed age distribution of breeding individuals
for (t in 1:nyears){
  for (i in 1:2){       # sex
    w[i,t,1:3] ~ dmulti(prAge[i,t,1:3], wT[i,t])
  } #i
  # females
  prAge[1,t,1] <- N[2,1,t] / (N[2,1,t] + N[4,1,t] + N[5,1,t])
  prAge[1,t,2] <- N[4,1,t] / (N[2,1,t] + N[4,1,t] + N[5,1,t])
  prAge[1,t,3] <- N[5,1,t] / (N[2,1,t] + N[4,1,t] + N[5,1,t])
  # males
  prAge[2,t,1] <- N[2,2,t] / (N[2,2,t] + N[4,2,t] + N[5,2,t] + I[t])
  prAge[2,t,2] <- N[4,2,t] / (N[2,2,t] + N[4,2,t] + N[5,2,t] + I[t])
  prAge[2,t,3] <- (N[5,2,t]  + I[t])/ (N[2,2,t] + N[4,2,t] + N[5,2,t] +
I[t])
} #t


# 2.3. Capture-recapture model (CJS model with multinomial likelihood)
for (k in 1:2){
  for (t in 1: (nyears-1)){
  marr[t,1:nyears,k] ~ dmulti(pr[t,1:nyears,k], rel[t,k])
  } #t
  # Define the cell probabilities of the m-arrays
  for (t in 1:(nyears-1)){
    # Main diagonal
    q[1,k,t] <- 1-p[1,k,t]
    q[2,k,t] <- 1-p[2,k,t]
    pr[t,t,k] <- phi[k,t] * p[1,k,t]
    # Further above main diagonal
    for (j in (t+2):(nyears-1)){
      pr[t,j,k] <- prod(phi[k,(t):j]) * q[1,k,t] * prod(q[2,k,(t+1):(j-1)])
* p[2,k,j]
    } #j
    # Below main diagonal
    for (j in 1:(t-1)){
      pr[t,j,k] <- 0
    } #j
  } #t
  # One above main diagonal
  for (t in 1:(nyears-2)){
    pr[t,t+1,k] <- phi[k,t] * phi[k,t+1] * q[1,k,t] * p[2,k,t+1]
  } #t
  # Last column: probability of non-recapture
  for (t in 1:(nyears-1)){
    pr[t,nyears,k] <- 1-sum(pr[t,1:(nyears-1),k])
  } #t
} #k


# 2.4. Dead-recovery model
for (k in 1:2){
  for (t in 1:(nyears-1)){
    marrD[t,1:nyears,k] ~ dmulti(prD[t,1:nyears,k], relD[t,k])
  } #t
  # Define the cell probabilities of the m-array
  for (t in 1:(nyears-1)){
    # Main diagonal
    prD[t,t,k] <- (1-s[1,k,t]) * r[k,t]
    # Further than three above main diagonal
    for (j in (t+3):(nyears-1)){
```

```
      prD[t,j,k] <- s[1,k,t] * s[2,k,t+1] * prod(s[3,k,(t+2):(j-1)]) * (1-
s[3,k,j]) * r[k,j]
    } #j
    # Below main diagonal
    for (j in 1:(t-1)){
      prD[t,j,k] <- 0
    } #j
  } #t
  # One above main diagonal
  for (t in 1:(nyears-2)){
    prD[t,t+1,k] <- s[1,k,t] * (1-s[2,k,t+1]) * r[k,t+1]
  } #t
  # Two above main diagonal
  for (t in 1:(nyears-3)){
    prD[t,t+2,k] <- s[1,k,t] * s[2,k,t+1] * (1-s[3,k,t+2]) * r[k,t+2]
  } #t
  # Last column: probability of non-recovery
  for (t in 1:(nyears-1)){
    prD[t,nyears,k] <- 1-sum(prD[t,1:(nyears-1),k])
  } #t
} #k


# 2.5. Probability of breeding success
# Define the multinomial likelihood
for (a in 1:3){
  for (t in 1:nyears){
    psy[a,t] ~ dbinom(eta[a,t], pst[a,t])
  } #t
} #a


# 2.6. Sex ratio data
for (i in 1:nsr){
  fchicks[i] ~ dbin(xi[agef.sr[i], year.sr[i]], tchicks[i])
}

# 2.7. Number of fledglings, given success
# Define the normal likelihood
for (i in 1:nchicks){
  yc[i] ~ dnorm(rho[agef.chicks[i], year.chicks[i]], sd=sigma.chicks)
}
})
```

References

Bijlsma, R.G., 1994. Ecologische atlas van de Nederlandse Roofvogels, 3. druk 1994. Schuyt, Haarlem.

Choquet, R., Lebreton, J.-D., Gimenez, O., Reboulet, A.M., Pradel, R., 2009. U-CARE: Utilities for performing goodness of fit tests and manipulating CApture-REcapture data. Ecography 32, 1071–1074.

de Valpine, P., Hastings, A., 2002. Fitting population models incorporating process noise and observation error. Ecol. Monogr. 72, 57–76.

de Valpine, P., Paciorek, C.J., Turek, D., Michaud, N., Anderson-Bergman, C., Obermeyer F., Wehrhahn Cortes C., Rodriguez, A., Temple Lang D., Paganin, S., 2020. NIMBLE User Manual. R package manual version 0.10.1.

de Valpine, P., Turek, D., Paciorek, C.J., Anderson-Bergman, C., Lang, D.T., Bodik, R., 2017. Programming with models: writing statistical algorithms for general model structures with NIMBLE. Journal of Computational and Graphical Statistics 26, 403–413.

Kéry, M., Schaub, M., 2012. Bayesian population analysis using WinBUGS - A hierarchical perspective, 1st ed. Academic Press, Boston.

Looft, V., 2017. Habichtjahre - Langzeitstudie zur Brutbiologie des Habichts verbunden mit der Suche nach den beeinflussenden Faktoren. Corax 23, 161–235.

Pradel, R., 1993. Flexibility in survival analysis from recapture data: handling trap-dependence. In: Lebreton, J.-D. (Ed.), Marked Individuals in the Study of Bird Population. Birkhäuser-Verlag, Basel, pp. 29–37.

Schaub, M., Kéry, M., 2022. Integrated population models. Theory and ecological applications with R and JAGS. Academic Press an imprint of Elsevier, London.

Williams, B.K., Nichols, J.D., Conroy, M.J., 2002. Analysis and management of animal populations. Modeling, estimation, and decision making. Academic Press, San Diego, USA.
